# Highly‐Specific Single‐Stranded Oligonucleotides and Functional Nanoprobes for Clinical Determination of Chlamydia Trachomatis and Neisseria Gonorrhoeae Infections

**DOI:** 10.1002/advs.202304009

**Published:** 2023-10-23

**Authors:** Ketan Dighe, Parikshit Moitra, Nivetha Gunaseelan, Maha Alafeef, Tor Jensen, Carla Rafferty, Dipanjan Pan

**Affiliations:** ^1^ Department of Pediatrics Centre of Blood Oxygen Transport & Hemostasis University of Maryland Baltimore School of Medicine Baltimore Maryland 21201 USA; ^2^ Department of Chemical & Biochemical Engineering University of Maryland Baltimore County Baltimore County Maryland 21250 USA; ^3^ Department of Biomedical Engineering The Pennsylvania State University University Park PA 16802 USA; ^4^ Department of Nuclear Engineering The Pennsylvania State University University Park PA 16802 USA; ^5^ Cancer Center at Illinois University of Illinois Urbana‐Champaign 405 N. Mathews Ave. Urbana IL 61801‐2325 USA; ^6^ Department of Family Medicine Carle Health 1818 E Windsor Rd. Urbana IL 61802 USA; ^7^ Department of Materials Science and Engineering The Pennsylvania State University University Park PA 16802 USA; ^8^ Huck Institutes of the Life Sciences 101 Huck Life Sciences Building University Park PA 16802 USA

**Keywords:** Chlamydia trachomatis, gold nanoparticles, lateral flow assay, Neisseria gonorrhoeae, point‐of‐care, single‐stranded oligonucleotides

## Abstract

Early detection of *Chlamydia trachomatis* (CT) and *Neisseria gonorrhoe*ae (NG) is the key to controlling the spread of these bacterial infections. An important step in developing biosensors involves identifying reliable sensing probes against specific genetic targets for CT and NG. Here, the authors have designed single‐stranded oligonucleotides (ssDNAs) targeting mutually conserved genetic regions of cryptic plasmid and chromosomal DNA of both CT and NG. The 5′‐ and 3′‐ ends of these ssDNAs are differentially functionalized with thiol groups and coupled with gold nanoparticles (AuNP) to develop absorbance‐based assay. The AuNPs agglomerate selectively in the presence of its target DNA sequence and demonstrate a change in their surface plasmon resonance. The optimized assay is then used to detect both CT and NG DNA extracted from 60 anonymized clinical samples with a clinical sensitivity of ∼100%. The limit of detection of the assays are found to be 7 and 5 copies/µL for CT and NG respectively. Furthermore, it can successfully detect the DNA levels of these two bacteria without the need for DNA extraction and via a lateral flow‐based platform. These assays thus hold the potential to be employed in clinics for rapid and efficient monitoring of sexually transmitted infections.

## Graphical abstract

*Chlamydia trachomatis* (CT) and *Neisseria gonorrhoeae* (NG) are two of the most prevalent sexually transmitted infectious microorganisms in the world. In this study, novel single‐stranded DNA (ssDNA) probes are designed to target genetic segments of Chlamydia CT and NG. These probes are then attached to gold nanoparticles (AuNPs) to create an absorbance‐based assay. The assay relies on complementary binding between the probes and the target gene sequences unique to CT or NG. The method is validated with clinical samples, achieving high sensitivity and specificity. Furthermore, the assay is adapted for point‐of‐care (POC) use on a lateral flow platform, offering user‐friendliness and rapid results without the need for complicated DNA extraction and purification steps.

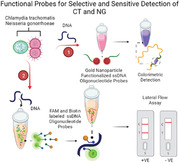

## 1 Introduction

Sexually transmitted infections (STIs) are a leading source of microbial infection worldwide, resulting in a substantial economic and healthcare burden.^[^ ^1^ ^]^ With 127 million and 87 million new infections each year, respectively, *Chlamydia trachomatis* (CT) and *Neisseria gonorrhoeae* (NG) are two of the most prevalent sexually transmitted infectious microorganisms in the world.^[^ ^2^, ^3^, ^4^, ^5^ ^]^ In the United States (U.S.), the Centers for Disease Control and Prevention (CDC) received reports of 1644416 chlamydia cases and 710151 gonorrhea cases in 2021 alone, making these STIs an ongoing epidemic.^[^ ^2^, ^3^, ^4^, ^5^ ^]^ Because many of these infections may exhibit no symptoms at all, the CDC has recommended universal annual Chlamydia screening in women aged 25 years old and younger and those older than 25 years of age with risk factors.^[^ ^6^, ^7^, ^8^ ^]^ While treatments are readily available for both these infections, if a patient does not get tested or fails to follow up with treatment, these infections can lead to long‐term complications such as urethritis in men and infertility, pelvic inflammatory disease, ectopic pregnancy, and chronic pelvic pain in women.^[^ ^6^, ^7^, ^8^, ^9^ ^]^ Furthermore, both these infections tend to develop antibiotic resistance over time. Antibiotic resistance can emerge quickly in NG compared to CT, and in 2020, it was estimated that at least 50% of gonorrhea infections were resistant to at least one antibiotic.^[^ ^10^, ^11^, ^12^ ^]^ Therefore, the increasing rates of chlamydia and gonorrhea mediated STIs pose a global public health issue.

Due to the high risk of CT and NG transmission and the lack of rapid and reliable testing, these STIs are routinely treated presumptively, resulting in both overtreatment and undertreatment.^[^ ^13^, ^14^, ^15^, ^16^, ^17^, ^18^, ^19^ ^]^ In some studies, the rates of overtreatment in emergency departments have been shown to be as great as 86%. That is, 86% of those receiving treatment are negative for these diseases.^[^ ^13^, ^14^, ^15^, ^16^, ^17^ ^]^ Concurrently, often as much as 50% of those who eventually receive positive results are not provided treatment at the time of testing or first visit to the clinic which may lead to transmission of the disease to others before treatment is sought.^[^ ^18^, ^19^ ^]^ Untreated cases may lead to long‐term morbidity as well as multiple medical and chronic conditions. Unnecessary use of antibiotics can also cause adverse effects and lead to increased antibiotic resistance in the community. While it is possible to improve treatment rates with intense post‐visit follow‐up procedures, this can be both costly and time intensive. Therefore, the timely diagnosis of CT and NG is essential for implementation of effective infection control measures.

The current gold standard for diagnosing chlamydia and gonorrhea infections are the established nucleic acid amplification tests (NAATs) that amplify and detect specific DNA sequences of CT and NG.^[^ ^20^, ^21^, ^22^ ^]^ Although these diagnostic approaches are excellent in terms of sensitivity and selectivity, they are expensive, time‐consuming, and cannot be deployed in a point‐of‐care (POC) settings (e.g., a physician's office or emergency department), hindering rapid results during the patient's visit.^[^ ^23^, ^24^, ^25^ ^]^ Therefore, the development of an accurate POC test for rapid and simple detection of these STIs is crucial, as it would enable timely treatment, prevent further spread, raise risk awareness, reduce costs, and advance healthcare in resource‐limited areas.^[^ ^26^, ^27^, ^28^, ^29^ ^]^ While several POC tests have been developed to identify CT and NG antigens using a lateral flow assay, these assays often underperform when compared to NAAT‐based tests and are not suitable for population screening in physician's offices.^[^ ^30^, ^31^ ^]^ Consequently, there is an ongoing need for improved assays and more rigorous evaluations to guide their potential application. Urgently, there is a need for a POC diagnostic method based on nucleic acid detection that combines usability, cost‐effectiveness, and the speed of isothermal amplification with high sensitivity and specificity.^[^ ^30^, ^31^ ^]^ Additionally, since CT and NG co‐infection are commonly encountered (up to 50% of the time) and present with similar symptoms, simultaneous identification and detection of both pathogens from suspected patient specimens are more efficient and cost‐effective.^[^ ^32^ ^]^ Currently most of the approved POC tests do not provide results for CT and NG on the same platform. Hence, the development of a rapid POC diagnostic assay capable of simultaneously detecting CT and NG is of utmost importance in addressing the current epidemic of these STIs.

In the development of a POC diagnostic assay for rapid detection of CT and NG, a crucial step involves the identification of reliable sensing probes that offer selective and sensitive detection of specific genetic targets in these bacterial agents.^[^ ^33^, ^34^ ^]^ Single‐stranded oligonucleotides (ssDNAs), which are fragments of nucleic acids, emerge as valuable tools in this process.^[^ ^35^, ^36^, ^37^, ^38^, ^39^, ^40^, ^41^, ^42^, ^43^, ^44^, ^45^, ^46^, ^47^ ^]^ They can be precisely designed to exhibit high specificity and affinity for highly conserved plasmid and genomic regions of CT and NG. These ssDNAs that target the conserved regions play a critical role in achieving specific and sensitive detection of the pathogens, enabling accurate identification even in scenarios where target genetic concentrations are low and minimizing the occurrence of false positives.^[^ ^40^, ^41^, ^42^, ^43^, ^44^ ^]^ Moreover, these nucleotide‐based sensing probes offer the advantage of broad applicability across different strains and geographic locations.^[^ ^40^, ^44^ ^]^ By targeting conserved regions, they can be utilized universally for diagnostic purposes, ensuring consistent and reliable results regardless of genetic variations among strains.^[^ ^40^, ^44^ ^]^ This feature enhances their utility and facilitates their integration into diagnostic workflows for widespread application. Additionally, the application of these probes extends beyond diagnosis and encompasses the selective detection of the target bacterial infection irrespective of their antibiotic resistance. By targeting conserved regions associated with resistance genes, they enable the efficient surveillance of CT and NG populations. This information is vital for understanding resistance patterns, informing treatment strategies, and combating the emergence and spread of drug‐resistant strain.

In this study, we designed a series of new oligonucleotide (ssDNA) probes targeting different genetic segments of CT and NG. The ssDNA probes were then conjugated on to the surface of gold nanoparticles (AuNPs) and their target binding efficacies were investigated. Our observations revealed that the ssDNA‐tagged AuNPs selectively aggregated in the presence of their target genes (DNA from either CT or NG), leading to an absorbance‐based assay. The principle governing the sensing mechanism of the absorbance‐based assay lies in the complementary binding between the novel oligonucleotide probes (ssDNA) and their targeted gene sequences unique to CT or NG. The ssDNA probes facilitate the aggregation of gold nanoparticles when they are attached to the nanoparticle surface and exposed to the specific target gene sequence. The close proximity seen between the ssDNA pair and the AuNPs can be attributed to the natural arrangement of the oligonucleotides, which includes thiol modifications (‐SH) at their 5′ and 3′ end. These modifications at 5′ and 3′ end promote the agglomeration of AuNPs in the presence of target gene or genetic material. Moreover, the process of aggregation leads to a noticeable increase in the diameter of nanoparticles, resulting in an increase of the extinction coefficient. As a result, there is a noticeable rise in absorbance at a wavelength of 523 nm, indicating the occurrence of AuNP aggregation upon interaction with the specific DNA sequence of interest. (Note: In this study, DNA has been isolated from the clinical samples using a commercially available DNA extraction kit). Moreover, when direct samples were introduced into the system together with an enzymatic extraction buffer, the AuNPs underwent aggregation, resulting in a wavelength shift from 523 to 630 nm. The confirmation of the sensing phenomena was achieved by the utilization of various bioanalytical techniques, including UV–Vis spectroscopy, transmission electron microscopy (TEM), Raman spectroscopy, and hyperspectral microscopy.

**Figure 1:**
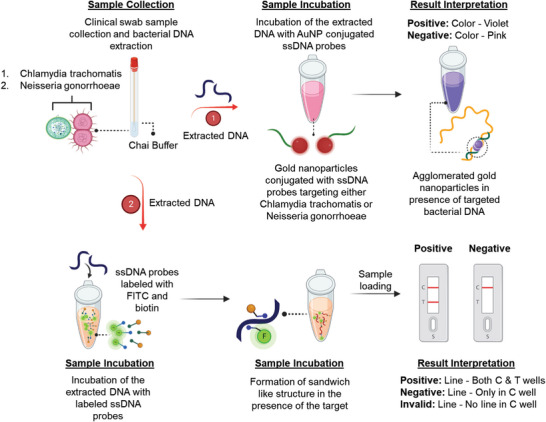
Schematic representation of two approaches based on changes in absorbance and lateral flow methods, utilizing novel oligonucleotide probes targeted toward *Chlamydia trachomatis* and *Neisseria gonorrhoeae*, for the clinical determination of chlamydia and gonorrhea. The addition of genetic material (bacterial DNA from either *Chlamydia trachomatis* or *Neisseria gonorrhoeae*) will result in a distinct change in absorbance. In the lateral flow assay, the presence of the target DNA will be indicated by a prominent test (T) line along with a control (C) line.

To validate sensing efficacy of the ssDNA‐tagged AuNPs in detecting the CT/NG target genes, we carefully standardized assay parameters such as incubation time, temperature, and reagent concentrations under different environmental conditions. Furthermore, we confirmed the capability of the developed assay by testing 60 anonymized clinical samples of CT and NG. The results indicated a high clinical sensitivity of 100% for CT detection and 100% for NG detection. Additionally, we evaluated the assay's specificity by conducting tests with a bacterial panel, achieving a limit of detection (LOD) of 7 copies µL^−1^ for CT and 5 copies µL^−1^ for NG. Moreover, we implemented the assay strategy on a lateral flow assay platform for the point‐of‐care (POC) detection of CT and NG. Excitingly, our results demonstrated that the lateral flow assay platform successfully differentiated between positive and negative samples within a timeframe of less than 10 min after sample addition, without requiring DNA extraction. Thus, the designed ssDNA‐based assays offer several advantages over other molecular tests currently used for CT and NG detection, including user‐friendliness, affordability, and portability POC diagnostics. Overall, we envision that a diagnostic tool based on the designed probes for CT and NG (**Figure** **1**) will not only combine the high accuracy, sensitivity, and specificity of nucleic acid (NA) based tests but will also be able to deliver results at the POC level during the patient's office or emergency room visit.

## 2 Results and Discussion

### 2.1 Selection of Target gene Sequence for *Chlamydia Trachomatis* and *Neisseria Gonorrhoea*e and Design of Single‐Stranded Oligonucleotides (ssDNAs)

**Figure 2:**
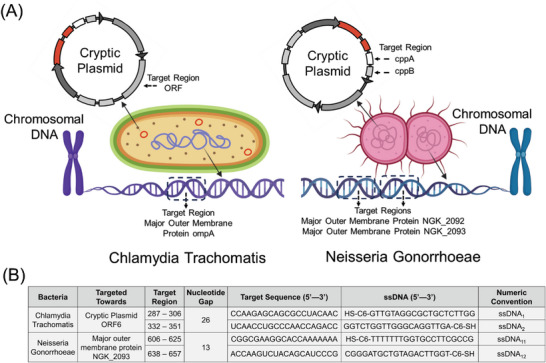
(A) Schematic representation of identified genetic targets in *Chlamydia trachomatis* and *Neisseria gonorrhoeae*. (B) Identified oligonucleotide sequences. ssDNA sequences identified toward the cryptic plasmid (ORF6) of *Chlamydia trachomatis*; ssDNA sequences identified toward the major outer membrane protein (NGK_2093) of *Neisseria gonorrhoeae*. The ssDNA sequences were subsequently modified with thiol groups (C6‐SH or HS‐C6) at either 5′ or 3′ end in order to conjugate to gold nanoparticles (AuNP).

*Chlamydia trachomatis* is an obligate intracellular bacterium that causes several diseases in humans; serovars A to C are linked with trachoma, serovars D to K infect the urogenital tract, and serovars L1 to L3 are typically more invasive and cause lymphogranuloma venereum (LGV). Although it appears that antibiotic‐resistance in *C. trachomatis* is not of immediate clinical^[^ ^48^ ^]^ significance, it may become resistant to macrolides through mutations in the 23S rRNA, rplD, and rplV genes, to fluoroquinolones through mutations in gyrA, parC, and ygeD genes, and to rifamycin and MDQA through mutations in rpoB and secY gene respectively.^[^ ^49^ ^]^ Hence, to design and develop highly selective and sensitive oligonucleotide (ssDNA) probes for *C. trachomatis*, we targeted the *ompA* (from the chromosome) and ORF6/pgp4 (from the plasmid) genes which are not responsible for antibiotic resistance and remains conserved across the strains. The highly conserved *ompA* gene which encodes the major outer membrane protein (MOMP) is a standard gene for all polymerase chain reaction (PCR) detection methods used in molecular laboratories and commercial tests.^[^ ^50^ ^]^ Furthermore, *C. trachomatis* serovar prediction also relies on nucleotide sequencing of the ompA gene. Apart from the highly conserved chromosome (>98% of similarity among strains), *C. trachomatis* naturally harbors a ≈7.5 kb plasmid.^[^ ^50^, ^51^ ^]^ The plasmid is also highly conserved among strains and possesses eight open reading frames (ORFs 1–8) those are known to be transcribed and translated.^[^ ^50^, ^51^ ^]^ Further, the analytical sensitivity of cryptic plasmid was found to be better than other genetic segments for CT. The reason behind the choice of ORF6 (**Figure** **2**A,B) over other ORFs for CT is that ORF6/pgp4 (transcriptional regulator 68 of virulence associated genes) presented the highest mean expression among all CT strains, followed by the virulence factor ORF5/pgp3 (also regulated by ORF6/pgp4).^[^ ^50^, ^51^ ^]^

However, it appears that *Neisseria gonorrhoeae* seems to have critical antimicrobial resistance and is evolving into a true “superbug” in the recent days.^[^ ^52^ ^]^ In addition to its function as outer membrane pore, the major outer membrane porin (PorB)^[^ ^53^ ^]^ expressed by *Neisseria gonorrhoeae* plays multiple essential roles during infection and its associated microbial resistance.^[^ ^53^ ^]^ Beside being the most prevalent protein on the outer membrane, PorB of *Neisseria gonorrhoeae*, is responsible to increase attachment, is then transported to the mitochondria of the host cell, and reduces the capacity of phagocytes to eradicate the bacterium.^[^ ^54^ ^]^ Resisting the effects of complement factors, controlling apoptosis, invading host cells, and involvement in antimicrobial resistance are further significant traits of porB.^[^ ^55^, ^56^ ^]^ Hence, to detect *Neisseria gonorrhoeae* strain, we decided to target chromosomal proteome of major outer membrane protein (MOMP) for NG (Figure 2A,B). Further to compare the selectivity of the ssDNAs generated by targeting the MOMP region, we have also included cryptic plasmid as the target gene for NG to design another set of ssDNAs.

Specific ssDNAs are then identified for these target genes comparing the binding energy and binding site disruption energies of all the ssDNAs as predicted by SOligo software. All the selected ssDNAs and their respective target sequences are shown in Table S1 (Supporting Information) (for CT), Tables S2 and S3 (Supporting Information) (for NG) for which the target binding energies were found to be optimal (at least ←8.0 kcal mol^−1^). Based on our previous research, an optimal balance between target binding energy and binding site disruption energy is necessary for the development of an effective oligonucleotide that can be used in a diagnostic assay.^[^ ^39^, ^40^, ^41^, ^42^, ^43^, ^44^ ^]^

### 2.2 Design and Synthesis of Gold Nanoparticles Capped with Designed Oligonucleotides

The ssDNAs were then selected as pairs and functionalized differentially either at their 5′ or 3′ ends. It has been hypothesized that when the 5′ end of the first ssDNA and 3′ end of the second ssDNA was thiol functionalized and conjugated with gold nanoparticles (AuNPs), it will give rise to increased agglomeration in presence of their target gene. It is further expected that the agglomeration among the gold nanoparticles will increase when the nucleotide gap between the two consecutive ssDNAs decreases. This aggregation phenomena among the nanoparticles in presence of their target gene were then studied based on the plasmon response of AuNPs.

**Figure 3:**
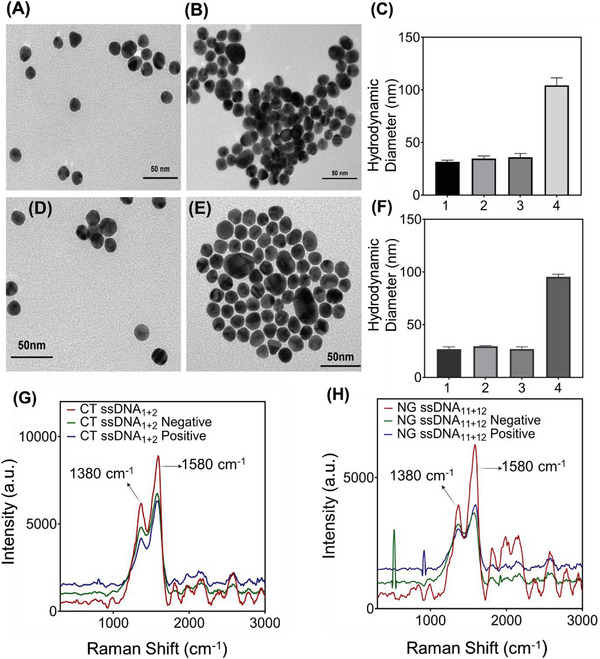
Transmission electron microscopy (TEM), Dynamic Light Scattering (DLS) and Raman spectroscopy data for CT targeted AuNP‐ssDNAs (A‐C &G) and NG targeted AuNP‐ssDNAs (D‐F &H). (A) ssDNA‐capped AuNPs targeted toward CT are individually dispersed with no visible aggregation in absence of CT DNA. (B) Visible aggregation of ssDNA‐capped AuNPs in presence of CT genomic DNA (0.5 ng µL^−1^). (C) Comparative changes in average hydrodynamic diameter of the ssDNA capped AuNP targeted toward CT (1. AuNP 2. ssDNA capped AuNP targeted toward CT, 3. ssDNA capped AuNP targeted toward CT in presence of NG and 4. ssDNA capped AuNP targeted toward CT in presence of CT). Data are presented as mean ± SD. Error bar indicates the measurements of the hydrodynamic diameter from three (*n* = 3) such independent experiments. (D) ssDNA‐capped AuNPs targeted toward NG are individually dispersed with no visible aggregation in absence of NG DNA. (E) Visible aggregation of ssDNA‐capped AuNPs in presence of NG genomic DNA (0.5 ng µL^−1^). (F) Comparative changes in average hydrodynamic diameter of the ssDNA capped AuNP targeted toward NG (1. AuNP 2. ssDNA capped AuNP targeted toward NG, 3. ssDNA capped AuNP targeted toward NG in presence of CT and 4. ssDNA capped AuNP targeted toward NG in presence of NG). Data are presented as mean ± SD. Error bar indicates the measurements of the hydrodynamic diameter from three (*n* = 3) such independent experiments. (G) Raman spectra for CT targeted AuNP‐ssDNAs in presence of CT genomic DNA (0.5 ng µL^−1^). (H) Raman spectra for NG targeted AuNP‐ssDNAs in presence of NG genomic DNA (0.5 ng µL^−1^).

In the present study, we designed four ssDNAs for CT (1 set of two targeting *ompA* gene and another set of two targeting ORF6 gene) and a total of fourteen ssDNAs for NG (4 sets of two each targeting major outer membrane protein and 3 sets of two each targeting cryptic plasmid) based on the principles stated above. These ssDNAs were then differentially functionalized with thiol moiety. While first ssDNA of each pair was functionalized at the 5′ end, the other ssDNA from that pair was functionalized at the 3′ end to increase their propensity of agglomeration in presence of their target gene (Figure 1). These differentially functionalized ssDNAs were then used to cap standard citrate stabilized gold nanoparticles (AuNPs) prepared from chloroauric acid and sodium citrate based on a previous protocol from our laboratory.^[^ ^39^, ^40^, ^41^, ^42^, ^43^, ^44^ ^]^ The formation of ssDNA‐conjugated AuNPs were confirmed using various characterization tools such as UV–vis absorbance spectroscopy, transmission electron microscopy (TEM), and nuclear magnetic resonance spectroscopy (NMR). TEM images showed that the ssDNA‐capped AuNPs are individually dispersed with no visible aggregation (**Figure** **3**A (CT) and Figure 3D (NG)). Figure 3C,F shows the average hydrodynamic sizes of the individual ssDNA capped AuNPs, which were found to be < 32 nm. Additionally, the surface plasmon bands of the ssDNA‐conjugated thiol‐stabilized AuNPs (λ_max_ at 523 nm) confirmed their formation (Figure S1, Supporting Information). The UV–vis band at 523 nm demonstrated that the nanoparticles maintained their properties after the conjugation process with thiolate ssDNA probes. NMR spectroscopy, on the other hand, showed the presence of aromatic protons, corresponding to guanosine and adenosine, in the ssDNA tagged AuNPs proving the successful conjugation of thiolate ssDNAs on the AuNP surface (Figures S2 and S3, Supporting Information). The ssDNA stabilized nanoparticles were further characterized with Raman spectroscopy. Figure 3G,H shows the Raman spectrum obtained from a fresh batch of ssDNA‐conjugated thiol‐stabilized AuNPs. The peak ≈1380 cm^−1^ may be assigned to the aromatic ring stretching modes, while the other intense peak at ≈1580 cm^−1^ can be assigned to the C = O stretching mode of both the carbonyls present in the structure and to the aromatic ring stretching vibrations of the nucleotides.^[^ ^57^ ^]^ It can further be said that these peaks are the combined features coming from different nitrogenous bases present in the ssDNA sequences where the major contributions may come from adenine and guanine, less significantly from thymine and the cytosine features may probably be hidden by the other (overall) signals.^[^ ^58^ ^]^

### 2.3 Standardization of ssDNA Capped Gold Nanoparticles for Selective and Sensitive Detection of CT and NG

Once the formation of ssDNA‐conjugated thiol stabilized AuNPs were confirmed, we optimized a variety of parameters for sensitive and selective detection of CT and NG. To determine the ideal ssDNA pairs, we first utilized DNA extracted from deidentified cervical swab samples as the input target sequence. In the case of CT, we utilized ssDNA_1+2_, ssDNA_3+4_, and ssDNA_1+2+3+4_ pairs capped with gold nanoparticles at 0.5 µM of ssDNA concentration individually to determine the relative sensitivity toward the target CT DNA sequence. The relative increase in absorbance at 523 nm was monitored, and it was observed that the ssDNA_1+2_ (targeting ORF 6) showed the maximum sensitivity toward the CT DNA among the three pairs (Figure S4, Supporting Information). This confirms that the ssDNAs targeting cryptic plasmid ORF6 gene of CT has the optimal sensitivity behind its detection. Moreover, it is intriguing for us that the two pairs of oligos did not provide a noticeable change in absorbance when the individual oligo pairs worked as expected when used in separation. The possible reasoning for this may be the differential folding of target DNA in presence of individual oligo pair with respect to the two pairs of oligos. This might happen in a 3D space that the target DNA fold in such a way in presence of two pair of oligos that restricts the expected increase in size or extinction co‐efficient of the gold nanoparticles. Due to which, we did not see a noticeable change in absorbance when it was compared with the change in absorbance with individual pair of oligos.

Similarly, in case of NG, we utilized ssDNA_1+2_, ssDNA_3+4_, ssDNA_5+6_, and ssDNA_7+8_ pairs capped with gold nanoparticles at 0.5 µM of ssDNA concentration to determine the relative sensitivity toward the target NG DNA sequence. The relative increase in absorbance at 523 nm was monitored; however, none of the ssDNA sequences showed optimum sensitivity toward NG DNA (Figure S5, Supporting Information). Subsequently, we predicted another set of ssDNA sequences targeting the same genetic segment but at a different location. Among the new set of ssDNA sequences, it was observed that ssDNA_11+12_ showed the maximum sensitivity toward the extracted NG DNA sequences (Figure S6, Supporting Information). This proves that the ssDNAs targeting major outer membrane protein of NG having the nucleotide gap of 13 between the pairs has the optimal sensitivity behind the NG detection.

Further to determine the optimum concentration of ssDNA conjugated to the AuNPs which may provide maximal sensitivity, we performed the experiment with CT targeted ssDNA_1+2_ conjugated AuNPs where the added ssDNA concentration was 0.2, 0.5 and 1 µM respectively. Two CT positive, negative and CT+NG positive samples were added to the suspensions, and it was observed that the 0.5 µM concentration of ssDNAs showed the maximum sensitivity for both CT and NG (Figure S7, Supporting Information).

As the current sensing principle lies in the hybridization of the complementary DNA sequences of ssDNA probes and bacterial DNA, the assay performance will be highly temperature dependent. Hence, to determine an optimum temperature for the assay, the ssDNA conjugated AuNPs were mixed their respective target DNAs and incubated either at room temperature (≈23 °C), 37 or 65 °C before measuring their change in plasmon response (absorbance). It was observed that 37 °C was the optimum temperature for CT targeted assay as it was providing higher change in absorbance for all the four positive samples tested herein compared to the assay where the AuNPs were incubated at room temperature. Although we found a higher change in absorbance at 65 °C for the positive samples, the relative higher change in absorbance for negative sample at this temperature led us to choose 37 °C as the preferred assay temperature (Figure S8, Supporting Information). Similar is the case for NG targeted assay where 37 °C provided higher change in absorbance for all the four positive samples with minimal change for negative sample (Figure S9, Supporting Information) led us to establish 37 °C as optimum assay temperature. Unfolding of separate DNA strands with increase in temperature from 23 to 37 °C might have led to better complementary binding of ssDNA probes with their target DNAs. However, increasing the temperature beyond an optimum value increased the non‐specificity of the ssDNA probes.

### 2.4 Chlamydia and Gonorrhea Detection from Anonymized Clinical Samples

After standardizing the ssDNA capped gold nanoparticles, the probes were employed to build the sensor platform for detection of CT and NG. We utilized a commercially available kit to extract DNA from deidentified cervical swab and urine samples as the input target sequence. We analyzed 60 samples (including both urine and cervical swab samples) out of which 15 were CT positive, 15 were both NG and CT positive, 15 were NG positive and 15 were both CT and NG negative. The positivity of the samples was validated using gold standard quantitative polymerase chain reaction (**Tables** **1**, **2**, **3**) (qPCR) and native agarose gel electrophoresis. Moreover, the corresponding Ct values of the deidentified clinical samples used in this study as obtained from qPCR are shown in Table S4 (Supporting Information) (for CT) and Table S5 (Supporting Information) (for NG). It is presumed that the absorbance of AuNPs will remain intact in absence of target DNA samples, however, it will be increased or shifted largely from 523 nm in presence of the target CT/NG DNA. The agglomeration of ssDNA‐capped AuNPs in the presence of bacterial DNA was further studied by Raman spectroscopy, UV–vis absorbance spectroscopy and transmission electron microscopic (TEM) studies. As expected, AuNPs capped with ssDNAs agglomerated in presence of respective DNAs from CT positive, both CT+NG positive, and NG positive samples whereas no agglomeration was observed for both CT+NG negative samples under TEM (Figure 3B,E; Figure S10, Supporting Information). In absence of target DNA, well distributed pattern of AuNPs was observed (Figure S10A and D for NG and Figure S10E and H for CT, Supporting Information), while in presence of target CT/NG DNA, the AuNPs were found to be largely aggregated (Figure S10B,C for NG and Figure S10F,G for CT, Supporting Information). Moroever the average hydrodynamic diameter of the ssDNA capped AuNPs increased largely with the addition of its target CT/NG DNA (Figure 3C,F). In absence of target CT/NG DNA, the AuNPs remain separated from each other. Due to this agglomeration of AuNPs in presence of target CT/NG DNA, either a bathochromic shift in absorbance from 523 to 630 nm or an increase in absorbance at 630 nm was observed (Figure S11, Supporting Information). This is to be noted that when we added extracted DNA samples to the AuNP suspension, there is only increase in absorbance at 523 nm with minimal change in absorbance at 630 nm (for Section 2.3 including Figures S4–S9, Supporting Information). However, the change in absorbance at 630 nm becomes prominent when the AuNP suspension was added with direct clinical samples in presence of enzymatic extraction buffer. Hence, for Figure S11 (Supporting Information), where we used direct samples to monitor the changes in absorbance, we followed the UV–vis absorption at 630 nm. The possible reasoning behind this might lie in the increase in diameter of gold nanoparticles in presence of target DNA that introduced dramatic and continuous increase in the extinction co‐efficient of the AuNPs leading to the increase in absorbance at 523 nm.^[^ ^59^ ^]^ However, with the addition of direct samples, the presence of enzymatic extraction buffer, led to further agglomeration among the AuNPs in presence of target CT/NG DNA, leading to their shift in absorbance from 523 to 630 nm and an increase in absorbance at 630 nm (Figure S11, Supporting Information). Both of this bathochromic shift and increase in absorbance at 630 nm is indicative of the formation of larger size gold nanoparticles only in presence of their respective target gene.^[^ ^39^, ^40^, ^41^, ^42^, ^43^, ^44^, ^59^ ^]^ Thus, UV–vis spectroscopy corroborated the observation obtained from TEM. Further, when the ssDNA conjugated AuNPs were investigated under Raman spectroscopy, we found a red shift in the intense Raman band at 1580 cm^−1^ in presence of their target gene only. However, there was no shift in Raman peak when a non‐specific DNA segment (i.e., negative sample) was added to the ssDNA conjugated AuNPs. The phenomena were found to be valid both for CT (Figure 3C) and NG (Figure 3F) DNAs. This indicates successful hybridization between the target CT/NG DNA and their complementary ssDNAs. It may further be concluded that during hybridization event, the complementary nucleotides bind with each other via hydrogen bonding where carbonyl (C = O) stretching frequency participated the most and hence we can observe a distinct red shift in the intense Raman peak at 1580 cm^−1^ by ≈20 cm^−1^.^[^ ^57^ ^]^

**Figure 4:**
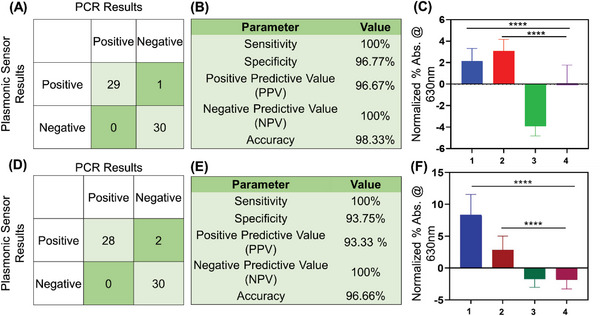
Chlamydia and Gonorrhea detection from sixty (*n* = 60) deidentified clinical samples. (A) Confusion matrix obtained from the demonstrated CT DNA targeting ssDNA conjugated AuNP based test. Results were benchmarked with the gold standard technique qPCR for the sixty (*n* = 60) deidentified clinical samples. (B) CT DNA targeting ssDNA conjugated AuNP based test parameters indicating the sensitivity, specificity, PPV, NPV and accuracy (*n* = 60). (C) Graphical representation of normalized percentage (%) absorbance change at 630 nm for the CT DNA targeted ssDNA conjugated AuNP based test. 1 is CT+ sample (*n* = 15); 2 is both CT+NG+ sample (*n* = 15); 3 is NG+ sample (*n* = 15) and 4 is both CT‐NG‐ samples(*n* = 15). Data are presented as mean ± SD. The one‐way ANOVA showed that the assay response was significantly different between positives and negatives. ****p‐value <0.0001 (D) Confusion matrix obtained from the NG DNA targeting ssDNA conjugated AuNP based test. Results were benchmarked with the gold standard technique qPCR from sixty (*n* = 60) deidentified clinical samples. (E) NG DNA targeted ssDNA conjugated AuNP based test parameters indicating the sensitivity, specificity, PPV, NPV and accuracy (n = 60). (F) Graphical representation of normalized percentage (%) absorbance change at 630 nm for NG DNA targeting ssDNA conjugated AuNP based test. 1 is NG+ sample (*n* = 15); 2 is both CT+NG+ sample (*n* = 15); 3 is CT+ sample (*n* = 15) and 4 is both CT‐NG‐ samples (*n* = 15). The one‐way ANOVA showed that the assay response was significantly different between positives and negatives. ****p‐value <0.0001.

**Figure** **4**A shows the confusion matrix of the tested clinical samples (using CT targeted AuNP‐ssDNAs) while the test results were benchmarked in the laboratory with the gold standard polymerase chain reaction (PCR) and quantitative polymerase chain reaction (qPCR). The PCR results were also validated using native agarose gel electrophoresis (Figures S12 and S13, Supporting Information). As mentioned above, a total number of 60 deidentified clinical samples have been tested using our platform, of which 29 were confirmed CT positive and 30 were confirmed negative cases. There was one case where a qPCR‐confirmed negative sample was misclassified as positive using our test (using CT targeted ssDNA). Figure 4B shows the accuracy, sensitivity, and specificity of our test as 98.3%, 100%, and 96.77% respectively. Figure 4C shows the normalized percentage changes, plotting the mean and standard deviation in absorbance at 630 nm for CT positive samples (CT+); both CT+NG positive samples (CT+NG+); NG positive samples (NG+) and both negative samples (CT‐NG‐). The percentage change in absorbance was found to be higher for CT positive samples and both CT and NG positive samples in comparison to the negative samples when tested with CT targeted ssDNA conjugated AuNPs. A one‐way repeated measures analysis of variance (ANOVA) statistical test was performed between the group to evaluate the assays response toward the various sample groups under investigation. The one‐way ANOVA showed that the groups showed a response that was significantly different with p<0.0001 for CT+ and CT+ NG+ when compared to negative samples (CT‐ and NG‐). However, the assay response was not significant in case of NG+ when compared to negative samples.

Similarly, a total number of 60 deidentified clinical samples were tested using NG targeted ssDNA conjugated AuNPs, of which 28 were confirmed as NG positive and 30 were confirmed as negative cases (Figure 4D). There were two cases where qPCR‐confirmed negative samples were misclassified as positive using our test (using NG targeted ssDNAs). Figure 4E shows the accuracy, sensitivity, and specificity of our test as 96.6%, 100% and 93.75% respectively. Figure 4F shows the normalized percentage change, plotting the mean and standard deviation in absorbance at 630 nm for NG positive samples (NG+), both CT and NG positive samples (CT+NG+), CT positive samples (CT+) and both negative samples (CT‐NG‐). As expected, the percentage change in absorbance was higher for NG positive samples and both CT+NG positive samples in comparison to negative samples. A one‐way ANOVA statistical test was performed between the group to evaluate the assays response toward the various sample groups under investigation. The one‐way ANOVA showed that the groups showed a response that was significantly different with p<0.0001 for NG+ and CT+ NG+ when compared to negative samples (CT‐ and NG‐). However, the assay response was not significant in case of CT+ when compared to negative samples. Moreover, the assays to detect CT and NG were also performed separately so that there is no confusion to selectively detect the target bacteria.

Thus, the current study utilizes the intrinsic optical properties of plasmonic AuNPs to demonstrate the targeting ability of the developed ssDNAs to selectively detect CT and NG positivity.

### 2.5 Validation through Darkfield Hyperspectral Microscopy

**Figure 5:**
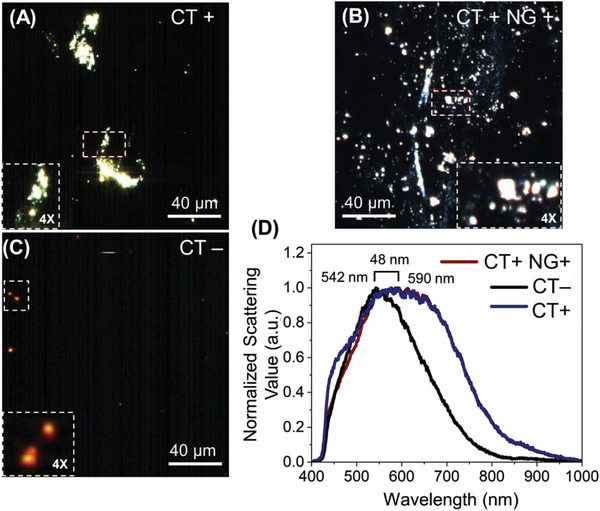
The hyperspectral imaging of the CT targeted ssDNA conjugated AuNPs in the presence of (A) CT+ (Ct = 25), (B) CT+ and NG+ (Ct = 30), and (C) CT‐ samples. (D) The hyperspectral data collected as a response to CT+, CT+ and NG+, and CT‐ samples from multiple positions of the image has been represented. The brackets show the distance between the peak maxima between the spectra obtained before and after the addition of the target DNA. The experiments were performed with experimental repeats of *n* = 5.

Hyperspectral imaging is a powerful technique that allows for the non‐destructive and label‐free analysis of materials and biological samples. In the context of confirming the binding of CT/NG genetic material, hyperspectral imaging can be used to visualize the interaction between CT/NG and their complementary ssDNA sequences. By analyzing the spectral signature of the sample, hyperspectral imaging can identify the specific wavelengths of light that are reflected by the sample.^[^ ^42^ ^]^ This information can be used to create an image that highlights the areas of the sample where the binding of CT/NG DNA is occurring. Additionally, hyperspectral imaging can provide information about the distribution of CT/NG within the sample, which can be useful for optimizing the binding conditions.^[^ ^42^ ^]^ Three samples have been evaluated including a sample with positive CT (CT+), a sample that has both CT and NG (CT+ and NG+), and a sample with negative CT (CT‐). We recorded the hyperspectral imaging collected using CT targeted ssDNA conjugated AuNPs as a response to CT+ (**Figure** **5**A), CT+ and NG+ (Figure 5B), and for a CT‐ (Figure 5C) sample. It has been observed that in both the samples with CT+, the AuNPs form an aggregate which has not been observed in the case of CT‐ sample. The hyperspectral peak of the CT+ samples showed a shift of ≈48 nm as compared to the CT‐ (Figure 5D) sample. Interestingly, the hyperspectral peak of the two samples with CT+ showed similar spectrum regardless of the presence of NG+ in the sample. This further confirms the specificity of the ssDNA probes used in the detection of CT in the sample.

**Figure 6:**
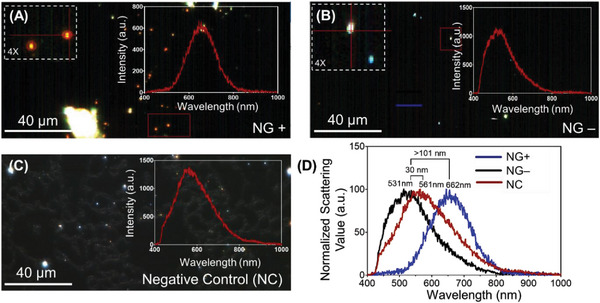
The hyperspectral imaging of the ssDNA‐AuNPs targeted toward NG in the presence of (A) NG+ (Ct = 27), (B) CT+ and NG‐ (Ct = 32) and (C) NG‐ samples. (D) The hyperspectral signature was collected as a response to NG+, CT+ and NG‐, and NG‐ samples from multiple positions of the image has been represented. The brackets show the distance between the peak maxima between the spectra obtained before and after the addition of the target DNA. The experiments were performed with experimental repeats of *n* = 5.

The same holds true for the ssDNA‐AuNPs targeted toward NG DNA. **Figure** **6** depicts the hyperspectral response of the AuNPs as a response to the samples containing NG genetic materials (NG+), samples lacking the NG DNA (NG‐) but having CT DNA (CT+), and negative control samples (CT‐ and NG‐). The AuNPs form aggregates in the presence of the NG DNA (Figure 6A), which was not observed in the case of NG‐ (Figure 6B) and the negative control (Figure 6C) samples. A significant peak shift in the hyperspectral signal was also observed (>101 nm) especially for NG+ sample compared to the negative samples (Figure 6D).

### 2.6 Direct Detection of CT and NG from Cervical Swabs and Urine Samples

**Figure 7:**
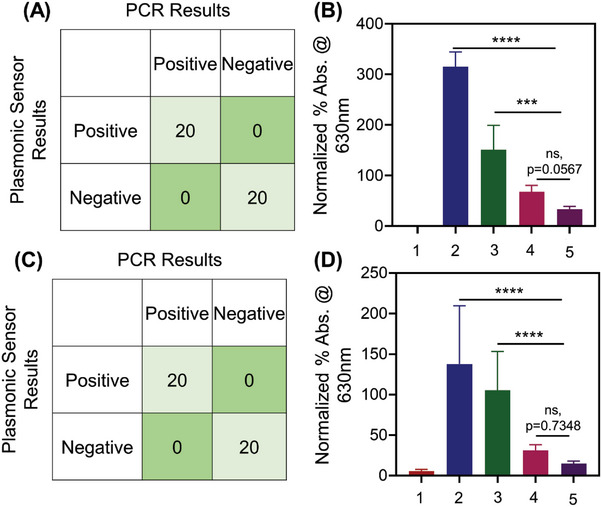
Chlamydia and Gonorrhea detection from forty (*n* = 40) deidentified clinical samples using CHAI buffer without any added purification steps. (A) Confusion matrix obtained from the studied ssDNA‐AuNPs targeting CT DNA. Results from forty (*n* = 40) deidentified clinical samples were validated with the gold standard technique qPCR. (B) Graphical representation of normalized percentage (%) absorbance change at 630 nm with CT targeted ssDNA‐AuNPs for 1. Control (water sample); 2. CT positive sample (*n* = 10); 3. both CT positive and NG positive sample (*n* = 10); 4. NG positive sample (*n* = 10) and 5. both CT and NG negative sample (*n* = 10). Data are presented as mean ± SD. The one‐way ANOVA showed that the assay response was significantly different between positives and negatives. ****p‐value <0.0001, ***p = 0.001, ns; *p* = 0.0567. (C) Confusion matrix obtained from the studied ssDNA‐AuNPs targeting NG DNA. Results from forty (*n* = 40) deidentified clinical samples were validated with the gold standard qPCR. (D) Graphical representation of normalized percentage (%) absorbance change at 630 nm with NG targeted ssDNA‐AuNPs for 1. Control (water sample), 2. NG positive sample (*n* = 10), 3. both CT positive and NG positive sample (n = 10), 4. CT positive sample (*n* = 10) and 5. both CT and NG negative sample (*n* = 10). Data are presented as mean ± SD. The one‐way ANOVA showed that the assay response was significantly different between positives and negatives. ****p‐value <0.0001, ns; *p* = 0.7348.

Generally, the nucleic acid‐based detection of bacterial species involves the isolation and purification of DNA that consists of multiple tedious steps. An ideal POC assay would be the one where the DNA targeting can be achieved in an easy and rapid manners. Toward this, we used the enzymatic DNA/RNA extraction buffer from CHAI added directly to the source media to detect CT and NG from the infected samples without any added purification steps. We analyzed 40 de‐identified samples (including both urine and cervical swab samples, N = 10 each for CT+, NG+, both CT and NG+, both CT and NG‐ (as validated using gold standard qPCR). **Figure** **7**A shows the confusion matrix of the tested clinical samples (using CT targeted ssDNA‐AuNPs) by benchmarking our test results to the gold standard qPCR. As indicated by the matrix, among the 40 deidentified clinical samples, 20 samples were confirmed CT positive and 20 were confirmed negative cases. The test also showed accuracy, sensitivity, and specificity of 100%, 100%, and 100% respectively. Figure 7B shows the normalized percentage changes, plotting the mean and standard deviation in absorbance at 630 nm for control water sample, CT+, both CT+ and NG+, NG+ and both CT‐ NG‐ samples. It was the percentage absorbance change was higher for CT+ and both CT+ NG+ sample but not for others when tested against CT targeted ssDNA‐AuNPs. A one‐way ANOVA statistical test was performed between the group to evaluate the assay response. The one‐way ANOVA showed that the groups showed a response that was significantly different with p<0.0001 for CT+ and p = 0.001 for CT+ NG+ when compared to negative samples (CT‐ and NG‐). However, the assay response was not significant (ns) with p = 0.0567 in case of NG+ when compared to negative samples. Similarly, Figure 7C shows the confusion matrix of the tested clinical samples (using NG targeted ssDNA‐AuNPs) by benchmarking our test results to the gold standard qPCR. Figure 7C validated the test results against gold standard qPCR and revealed good agreement with accuracy, sensitivity, and specificity of 100%, 100% and 100% respectively. Figure 7D shows the normalized percentage changes, plotting the mean and standard deviation in absorbance at 630 nm for control water sample, NG+, both CT+ and NG+, CT+ samples and both CT‐ and NG‐ samples. It was observed that the percentage absorbance change was higher for NG+ and both CT+ and NG+ samples but not for others when tested with NG targeted ssDNA conjugated AuNPs. A one‐way ANOVA statistical test was performed between the group to evaluate the assay response. The one‐way ANOVA showed that the groups showed a response that was significantly different with p<0.0001 for NG+ and CT+ NG+ when compared to negative samples (CT‐ and NG‐). However, the assay response was not significant with p = 0.7348 in case of CT+ when compared to negative samples. Further, it was observed from the UV–vis spectra that shift in absorbance to 630 from 523 nm was maximum for cervical swab samples compared to the urine samples (Figure S11, Supporting Information). This may entail the comparative less aggregation of the AuNPs in urine samples compared to the cervical swab samples.

### 2.7 Sensitivity and Specificity of the Demonstrated Absorbance‐Based CT/NG Assay

**Figure 8:**
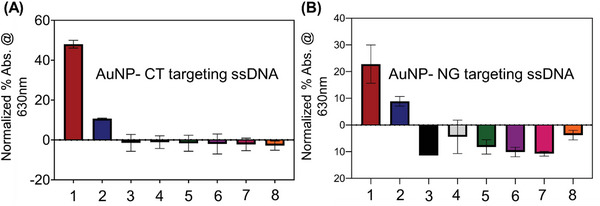
(A) Specificity study of CT targeting ssDNA‐AuNPs in presence of 1. Chlamydia trachomatis (Ct = 25), 2. Chlamydia trachomatis and Neisseria gonorrhoeae (Ct = 30), 3. Staphylococcus aureus, 4. Acinetobacter baumannii, 5. Escherichia coli, 6. Bacillus subtilis, 7. Streptococcus mutans and 8. negative control sample (water). (B) Specificity study of NG targeting ssDNA‐AuNPs in presence of 1. Neisseria gonorrhoeae (Ct = 27), 2. Chlamydia trachomatis and Neisseria gonorrhoeae (Ct = 32), 3. Staphylococcus aureus, 4. Acinetobacter baumannii, 5. Escherichia coli, 6. Bacillus subtilis, 7. Streptococcus mutans and 8. negative control sample (water). Data are presented as mean ± SD. Error bar indicates the measurements from three (*n* = 3) such independent experiments.

Encouraged by the positive results obtained above, we then tested the cross interference of the assay using a specific concentration of DNA obtained from fresh bacterial cultures (measured at OD600)^[^ ^60^ ^]^ of *Staphylococcus aureus*, *Acinetobacter baumannii*, *Escherichia coli*, *Bacillus subtilis* and *Streptococcus mutans*. As shown in **Figure** **8**, the normalized absorbance changes at 630 nm indicated that the designed ssDNA‐AuNP assay is highly selective toward their target sequence (either CT or NG). Therefore, the results demonstrated that the developed ssDNA‐AuNP assay had little to no cross‐reactivity with genetic material obtained from other infectious pathogens. Next, to establish the sensitivity, we utilized NG genomic DNA (ATCC 700825DQ) and determined the analytical limit of detection (LOD) of the developed assay for gonorrhea. Briefly, the assay was tested using the genomic DNA with serial 10‐fold dilutions with concentrations ranging from 10^5^ copies µL^−1^ to 10 copies mL^−1^ as shown in Figure S12 (Supporting Information). 10 µL of serially diluted sample was added to 200 µL of ssDNA_11+12_ targeting NG. It was observed that the test provided a detectable signal even when the NG genomic DNA concentration was as low as 10 copies µL^−1^. However, the normalized absorbance change was stable up to 100 copies mL^−1^. To calculate the analytical limit of detection of the NG assay, we used the equation $LOD\;=\;3.3*(\frac{Sy}{S})$. The LOD was determined to be 5 copies µL^−1^. It is worthwhile to mention that the LOD was calculated for the final sample (DNA mixed with the ssDNA labeled AuNP). Similarly, for chlamydia, we utilized the CT genomic DNA (ATCC VR‐901BD) to calculate the LOD which was found to be 7 copies µL^−1^ (Figure S13, Supporting Information).

### 2.8 Lateral Flow Assay Based Detection of CT and NG

**Figure 9:**
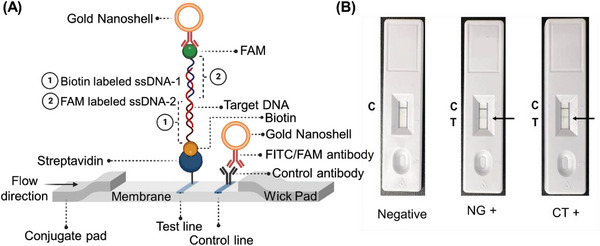
(A) Schematic representation of the operating principle of the lateral flow assay. The test line appears only in presence of the target either CT or NG bacterial DNA. The test line immobilized with streptavidin captures the biotinylated ssDNAs and the FAM conjugated ssDNAs capture the anti‐FAM coated gold nanoshells. (B) Representative lateral flow strips tested with negative sample (water), NG positive (Ct = 17) and CT positive samples (Ct = 24).

It is fundamentally necessary to develop a point‐of‐care diagnostic test with reasonable specificity and sensitivity, which can be implemented immediately without the need for any supporting equipment. Thus, to make the ssDNA‐AuNP assay an effective POC assay, we used commercially available lateral flow strips (Nanocomposix) to develop a functional lateral flow assay (LFA) for CT/NG detection.^[^ ^61^ ^]^ As the study confirmed the targeting capabilities of the novel ssDNA probes and provided us confidence in the development of lateral flow assays, we were able to move forward with the design of lateral flow assay as we had previously done for SARS‐CoV‐2.^[^ ^44^ ^]^ Accordingly, we modified the 5′‐ end of one of the optimized ssDNAs with biotin and 3′‐ end of the other with 6‐carboxyfluorescein (6‐FAM) and included them directly in the lateral flow assay. For our assay, total DNA was isolated using CHAI enzymatic DNA/RNA extraction buffer. 10X buffer was used for swab samples, whereas 1X buffer was used for urine samples. As a proof‐of‐concept experiment, we utilized the total DNA isolated from random clinical sample. The sample used in this experiment had a Ct value of 17 for NG and a Ct value of 24 for CT as determined using gold standard qPCR. Briefly, 10 µL of the isolated DNA sample (either CT or NG) was incubated separately with 10 µL AuNP‐ssDNA_11+12_ (in case of NG) and AuNP‐ssDNA_1+2_ (in case of CT) at 37 °C. The incubation ensures unfolding of target DNA and optimum hybridization with the ssDNA strands. The resulting mixture (20 µL) along with running buffer (105 µL) was then added to the lateral flow strip. The result is available after ≈10–15 min and can be read with the naked eyes. As the sample flows through the test strips, in the presence of the target DNA, FAM/biotin labeled ssDNA probes bind to their complementary target sequence for either CT or NG bacterial DNA. The test line (T) immobilized with streptavidin, captures this sandwich assembly having the biotin labeled ssDNA. The anti‐FAM antibody coated gold nanoshells are then attracted by the FAM‐labeled ssDNA leading to the formation of a faint red T line (**Figure** **9**A). The control species antibody present at the control line (C) captures the remaining unreacted anti‐FAM antibody‐coated gold nanoparticles, which confirms that the lateral flow system is working properly. The LFA results are represented in Figure 9B. However, in absence of the target DNA, the labeled ssDNAs cannot bind to any target and hence the bridged assembly will not form. Consecutively, anti‐FAM antibody‐coated gold nano shells will not stay on the test line and cannot show any test band (Figure 9B). Thus, the developed platform can differentiate between CT/NG positive and negative samples without the need for any DNA extraction or amplification.

## 3 Conclusion

Treatment for *Chlamydia trachomatis* (CT) and *Neisseria gonorrhoeae* (NG) is typically presumptive because of non‐availability of accurate tests or delayed test results, leading to over‐ and under‐treatment. At the ED settings, empiric treatment decisions are made leading to an overtreatment and undertreatment of patients with confirmed CT and NG cases. Untreated cases may lead to long‐term morbidity as well as multiple medical complications and adverse sequalae. Unnecessary use of antibiotics can also cause adverse effects and lead to increased antibiotic resistance in the community. Rapid and accurate identification of CT and NG is therefore essential for the timely implementation of infection control measures and to enable appropriate use of antibiotics dosage.^[^ ^62^, ^63^, ^64^ ^]^ The study, detailed herein, aims to address all these issues. Single‐stranded oligonucleotides (ssDNAs) are designed herein with high specificity and affinity for CT and NG. The targeted gene sequences are conserved among different strains of CT and NG and less prone to antibiotic resistance leading to minimal chance for false negativity.

Unlike the conventional protein/antigen based lateral flow assay, we have developed a nucleic acid based reproducible lateral flow assay that does not require any additional step of bacterial DNA extraction and purification. The assay procedure, described herein, is much simpler than many currently available diagnostic workflows and hence easily adaptable for rapid clinical diagnosis of CT and NG. Due to the simplicity of the developed assay, the efficiency of the lateral flow tests can be tested in a physician's office during the visit of the patient. The study has been fully validated first with gold nanoparticles conjugated with designed thiolated single stranded oligonucleotides added with DNA extracted from the infected clinical samples. Later, the assay was also standardized with direct clinical samples without the extraction and purification of bacterial DNA. The limit of detection of the CT and NG targeted assay was found to be 7 and 5 copies µL^−1^ respectively. Further, the assay is found to have minimal or no cross‐interference from other bacterial species. Thus, we demonstrated here a unique CT/NG assay for rapid and accurate clinical determination of STI where the sensing principle lies in the complementary binding of the newly designed oligonucleotide probes with their target gene sequence. This molecular assay can further be used to reflect the criticality of the disease as increased presence of target CT or NG genetic segment in the clinical sample will entail the severity of infection. Due to the choice of target gene sequence during the design of oligonucleotides, we envision that this developed test will be minimally affected by the antibiotic resistant bacterial samples and the chances for false negativity will be less. Now toward the commercialization of this assay, we are further evaluating the performance of this technology in a large pool of clinical samples. The efficiency of the lateral flow tests will then be tested in a physician's office during the visit of the patient.

## 4 Experimental Section

### Materials

All the chemicals were purchased from reputable commercial vendors and used without any further purification steps. Custom‐made oligonucleotides (ssDNAs) functionalized with thiol moieties either at 5′– or 3′– ends were purchased from Sigma–Aldrich (Saint Louis, MO). Custom‐made oligonucleotides (ssDNAs) functionalized with biotin and FAM were purchased from Integrated DNA Technologies (Coralville, IA). Polymerase chain reaction (PCR) primers were also obtained from Sigma Aldrich (Saint Louis, MO). PCR and qPCR reagents were obtained from New England Biolabs (Ipswich, MA) unless otherwise mentioned. Lysis Buffer and DNA isolation kit (Purelink Microbiome DNA Purification kit) were ordered from Invitrogen (Carlsbad, CA). One step enzymatic DNA/RNA extraction buffer 1X and 10X was ordered from CHAI (Santa Clara, CA). AmpliDetect – Nucleic Acid Lateral Flow Assay (NALFA) strips were custom ordered from nanoComposix (San Diego, CA). De‐identified *Chlamydia trachomatis* and *Neisseria gonorrhoeae* samples (cervical/vaginal swabs (women) and urine (men)) were obtained from Boca Biolistics (Pompano Beach, FL) and Carle Foundation Hospital (Urbana, IL). Genomic DNA was obtained from American Type Culture Collection (ATCC – Manassas, VA) . Quantitative Genomic DNA from Chlamydia trachomatis LGV Serovar I strain 440 VR‐901BD and Quantitative Genomic DNA from Neisseria gonorrhoeae 700825DQ.

### Design of Single‐Stranded Oligonucleotides (ssDNA) and Polymerase Chain Reaction (PCR) Primers

The single stranded oligonucleotides were designed based on the previously described approach using the SOligo software.^[^ ^39^, ^40^, ^41^, ^42^, ^43^, ^44^ ^]^ The primer sequences used for PCR along with their melting temperatures (Tm°) are listed below.

**Table 1:** Identified primer sequences along with their melting temperature (Tm°) used for polymerase chain reaction.

Primer	Sequence	Tm°
CT–LP	AGCGGTAAAACTGCTTACTGGTCA	66.6
CT–RP	TCGCCTTTTCTAGCGGCCAA	70.8
NG–LP	ACGGCGAATCTTACCACGTT	65.5
NG‐RP	TGCCATCTTGTTGTTGTGCG	68.3

### Citrate‐Stabilized Gold Nanoparticle (AuNP) Synthesis

The citrate capped AuNP were synthesized according to our previously reported protocol. Briefly, 8.5 mg of tetra chloroauric (III) acid trihydrate (HAuCl_4_·3H_2_O) was dissolved in 95 mL of deionized (DI) water. The resultant mixture was then transferred to a 200 mL round bottom flask with a reflux condenser submerged in an oil bath and heated to boiling while being magnetically stirred. 5.0 mL of sodium citrate solution (1%, w/v) was then quickly added to the flask and the solution was kept boiling and stirring for 30 min until its color turned wine red. The solution was cooled, and the final product was kept at room temperature and in the dark for future use.

### Functionalization of AuNP with ssDNA

2 mL of citrate stabilized AuNPs were taken and treated with ssDNAs at different concentrations (0.2 µM, 0.5 µM & 1.0 µM). Additionally, 0.5 mM of tris(2‐carboxyethyl) phosphine (TCEP) was added to the solution. The resulting mixture was magnetically stirred at room temperature for 2 h. The ssDNA‐AuNP solution was stored at 4 °C until further use.

### Storage and Handling of De‐Identified Patient Samples

The de‐identified clinical samples were obtained from Boca Biolistics and Carle Foundation Hospital. Permission was obtained from the Institutional Review Board (IRB) for safe handling of these contagious samples. All the studies with clinical samples were approved by the IRB of the University of Maryland Baltimore (UMB), under study protocol HP‐00094565. 60 de‐identified clinical (15 *Chlamydia* positive, 15 *Neisseria* positive, 15 both *Chlamydia and Neisseria* positive and 15 negative) vaginal/cervical swabs, urine samples confirmed using qPCR. The samples were stored at − 80 °C until further use.

### Bacterial Culture

*A. baumannii* and *S. aureus* were cultured in Tryptic Soy broth overnight at 37 °C under aerobic conditions with shaking at 200 rpm. *E. coli* was grown in Luria‐Bertani (LB) broth at 37 °C under aerobic conditions. *S. mutans* was cultured in a solution containing brain heart infusion (BHI).

### DNA Isolation using a Commercial DNA Extraction Kit

Total DNA was isolated from the 60 de‐identified clinical samples using the PureLink™ Microbiome DNA purification kit's (Invitrogen, cat. no. A29790) manufacturer's protocol.

### One‐step Enzymatic DNA Extraction

Total DNA was extracted from 40 de‐identified clinical (10 *Chlamydia* positive, 10 *Neisseria* positive, 10 both *Chlamydia and Neisseria* positive and 10 both *Chlamydia and Neisseria* negative) vaginal/cervical swabs, urine samples using CHAI enzymatic DNA/RNA extraction buffer. 10X buffer was used for swab samples, whereas 1X buffer was used for urine samples. Briefly, 20 µL of enzymatic DNA buffer was added to 180 µL of the liquid sample. The resulting mixture was then completely mixed by vortexing for 15 s and then incubated at room temperature for 15 min. The sample was then further incubated at 98 °C for additional 5 min. The resulting solution was analyzed using Thermo Scientific™ NanoDrop™ OneC Microvolume UV–vis Spectrophotometer for amount of total DNA present.

### Protocols for PCR and qPCR. Polymerase Chain Reaction (PCR)

The Polymerase Chain Reaction was set up as per manufacturer's (NEB – Taq 2X Master Mix M0270) protocol. Briefly, 1 µL each of 10 µM of forward and reverse primers, 5 µL of isolated/extracted DNA, 25 µL of *Taq* 2X master mix and 18 µL of nuclease‐free water were added to a PCR tube. All of the liquid was collected at the bottom of the tube following a gentle mixing of the reaction and a quick spin. The PCR tubes were then transferred to an Applied Biosystems™ MiniAmp™ Thermal Cycler with block preheated to 95 °C. The following thermocycling conditions were used to amplify the DNA:

**Table 2:** Protocol for polymerase chain reaction (PCR) and the thermocycling conditions used in the process.

PCR step	Temperature	Time
Initial Denaturation	95 °C	30 s
45 Cycles	95 °C	30 s
	68 °C	150 s (2:30 mins)
Final Extension	68 °C	5 min
Hold	4 °C	∞

The amplified products obtained were stored at – 20 °C until further use.

### Quantitative Polymerase Chain Reaction (qPCR)

The quantitative polymerase chain reaction (qPCR) was set up as per manufacturer's (NEB – Luna Universal qPCR Master Mix Protocol M3003) protocol. Briefly, 0.5 µL each of 10 µM of forward and reverse primers, 2 µL of isolated/extracted DNA, 10 µL of *Luna universal qPCR* master mix and 7 µL of nuclease‐free water were added to a 96‐well PCR plate. All of the liquid was collected at the bottom of the well following a gentle mixing of the reaction and a quick spin. The PCR plate was then transferred to an Azure Cielo qPCR System. The following thermocycling conditions were used to amplify the DNA:

**Table 3:** Protocol for quantitative polymerase chain reaction (qPCR) and the thermocycling conditions used in the process.

PCR step	Temperature	Time	Cycles
Initial Denaturation	95 °C	60 s	1
Denaturation	95 °C	15 s	45
Extension	60 °C	30 s	
Melt Curve	60–95 °C	various	1

### Agarose Gel Electrophoresis

The amplified products from PCR were also analyzed using a 2% agarose gel. Briefly, 2 grams of agarose were added to 1X TBE buffer and heated in a microwave. 5 µL of ethidium bromide was added to the resulting mixture and thoroughly mixed. The mixture was then added on to a cassette and allowed to be solidified. 100 bp DNA ladder; 1 kb DNA ladder and amplified products were then loaded into different lanes. The gel was run at 80 kV for 60 mins and imaged with Biorad GelDoc.

### UV–vis Spectroscopy

For absorbance measurement, 10 µL of DNA sample was mixed with 200 µL of AuNPs conjugated with ssDNA_1+2_ targeting CT and AuNPs conjugated with ssDNA_11+12_ targeting NG. The total DNA concentration was determined using Thermo Scientific™ NanoDrop™ OneC Microvolume UV–vis Spectrophotometer. The absorbance spectra for the assay with 96‐well plates were recorded on Biotek Synergy Neo2Microplate Reader both for end point, kinetic, and spectral analyses. Each of the experiments were repeated at least three times and an average of these spectra were presented. To standardize the assays, two of the samples from each category were selected randomly. The absorbance spectra were then normalized. The highest absorbance value was chosen for each spectrum, and then we divided the absorbance values by that number. The normalized data was then compared to standardize the assay parameters, regardless of the details of the experiment.

### Measurement of Hydrodynamic Diameter using Dynamic Light Scattering (DLS)

The average hydrodynamic diameter of the gold nanoparticles functionalized with the antisense oligonucleotides before and after the addition of DNA were monitored on a Malvern Nano‐S Zetasizer. The particles were diluted to an optimum extent before each measurement and multiplied by the dilution factor to obtain the results.

### Transmission Electron Microscopy

A 20 µL solution of the nanoparticles before and after the addition of target DNA was added on top of a carbon‐coated copper grid (400 mesh). This was allowed to stay for ≈10 min before being removed with a filter paper and imaged under a transmission electron microscope (FEI tecnai T12). The tungsten filament with 80 kV accelerating voltage was used for the investigations.

### Raman Spectroscopy

Raman spectra were recorded with an inVia confocal Raman microscope (Renishaw) at a laser excitation wavelength of 532 nm (power 1%, grating 1200 l/nm). A 50X objective lens was used for data collection with Raman shift ranging from 500 to 1800 cm^−1^. All the spectra were obtained by averaging at least 10 individual spectra obtained with an exposure time of 10 s. Renishaw 51P859 detector was used for these experiments.

### Hyperspectral Imaging

5 µL of each sample (CT/NG ssDNA conjugated AuNPs (20 µL) were mixed with target DNA (5 µL)) has been deposited on a glass slide and covered with a coverslip for the mixture to spread evenly. The slides were ready for analysis using hyperspectral microscopy. Using a dark‐field optical microscope (CytoViva), the samples were imaged. First, the system focuses the light path and illumination directly into the condenser, fixing the light path's geometry from the light source to the condenser's entrance slit. This focuses the maximum photon density on the sample, enabling an enhanced high signal‐to‐noise ratio. The enhanced dark‐field hyperspectral imaging was captured at 60X amplification using an oil objective. The hyperspectral signal had been recorded by first capturing the dark current image that was used as a background signal. The data was collected using a wide light spectrum ranging from 400 to 1000 nm. The scan was run for half of the field of view and the resultant multidimensional image has been further analyzed.

### Nuclear Magnetic Resonance Spectroscopy

The ^1^H NMR spectra were recorded on a Bruker 600 MHz spectrometer.

### Details of AmpliDetect – Nucleic Acid Lateral Flow Kit (NALF)

The nanoComposix AmpliDetect‐Nucleic Acid Lateral Flow Kit (ADLF‐25T) was used in this work. The AmpliDetect assay from nanoComposix is a universal detection method for nucleic acid sequences and other molecules labeled with fluorescein (FITC/FAM) and biotin. The technology is based on lateral flow sandwich assay using the gold nanoshells for enhanced sensitivity. The reaction was performed using two ssDNAs: one labeled with 6‐FAM, and another labeled with biotin. In presence of target DNA, once the DNA‐DNA hybrid bridge is formed and the resulting mixture is added to the sample port along with the running buffer. On the conjugate pad, the anti‐FAM/FITC‐gold nanoshell conjugate captures the DNA‐DNA hybrid. The conjugate‐sample complex travels up the strip, and the streptavidin test line recognizes and captures the biotin. The control line of secondary antibody binds any residual conjugate and validates the assay.

### Statistics Analysis

Statistical data were analyzed via GraphPad Prism 9.5.1 All bar plots were presented as mean ± standard deviation (SD). All X‐Y plots were presented using the mean value obtained after repeated measures. Statistical analysis was performed using a one‐way repeated measures analysis of variance (ANOVA) to evaluate the assays response toward the various sample groups under investigation. Differences between experimental groups and control groups were considered statistically significant with a p‐value < 0.0001. The statistical analyses and sample size applied for each experiment were indicated in the figure legends.

## Conflict of Interest

Prof. Dipanjan Pan is the founder/cofounder of three University based start‐ups. None of these entities, however, supported this work.

## Supporting information

Supporting Information

## Data Availability

The data that support the findings of this study are available in the supplementary material of this article.
