# Supplementary material for: Highly‐Specific Single‐Stranded Oligonucleotides and Functional Nanoprobes for Clinical Determination of Chlamydia Trachomatis and Neisseria Gonorrhoeae Infections
Source: Adv Sci (Weinh). 2023 Oct 23;10(36):2304009. doi: 10.1002/advs.202304009 (PMC10754082; doi:10.1002/advs.202304009)
Supplement: Supplementary file 1 — Supporting Information [file ADVS-10-2304009-s001.pdf]

ADVANCED SCIENCE
Open Access

Supporting Information

for *Adv. Sci.*, DOI 10.1002/advs.202304009

Highly-Specific Single-Stranded Oligonucleotides and Functional Nanoprobes for Clinical Determination of Chlamydia Trachomatis and Neisseria Gonorrhoeae Infections

*Ketan Dighe, Parikshit Moitra, Nivetha Gunaseelan, Maha Alafeef, Tor Jensen, Carla Rafferty and Dipanjan Pan**

Supporting Information

Highly-specific Single-stranded Oligonucleotides and Functional Nanoprobes for Clinical Determination of Chlamydia trachomatis and Neisseria gonorrhoeae Infections

Ketan Dighe,[Ŧ,b,d,e] *Parikshit Moitra,*[Ŧ,a,b] *Nivetha Gunaseelan,*[b,d,e] *Maha Alafeef,*[a,b,d] *Tor Jensen,*[f] *Carla Rafferty*[g] *and Dipanjan Pan*[* a,b,c,d,e,h]

[a] Department of Nuclear Engineering, The Pennsylvania State University, University Park, PA 16802, USA

[b] Department of Pediatrics, Centre of Blood Oxygen Transport & Hemostasis, University of Maryland Baltimore School of Medicine, Baltimore, Maryland 21201, USA

[c] Department of Materials Science and Engineering, The Pennsylvania State University, University Park, PA 16802, USA

[d] Department of Chemical & Biochemical Engineering, University of Maryland Baltimore County, Baltimore County, Maryland 21250, USA

[e] Department of Biomedical Engineering, The Pennsylvania State University, University Park, PA 16802, USA

[f] Cancer Center at Illinois, University of Illinois Urbana-Champaign, 405 N. Mathews Ave. Urbana, IL 61801-2325, USA

[g] Department of Family Medicine, Carle Health, 1818 E. Windsor Rd. Urbana, IL, 61802, USA

[h] Huck Institutes of the Life Sciences, 101 Huck Life Sciences Building, University Park, PA 16802, USA

Email of the corresponding author: dipanjan@psu.edu

Ŧ Author of equal contributions

Table of Contents	Page number
Figure S1	3
Figure S2	4
Figure S3	5
Figure S4	6
Figure S5	7
Figure S6	8
Figure S7	9

Figure S8	10
Figure S9	11
Figure S10	12-13
Figure S11	14
Figure S12	15
Figure S13	16
Figure S14	17
Figure S15	18
Table S1	19
Table S2	19
Table S3	19
Table S4	20
Table S5	21

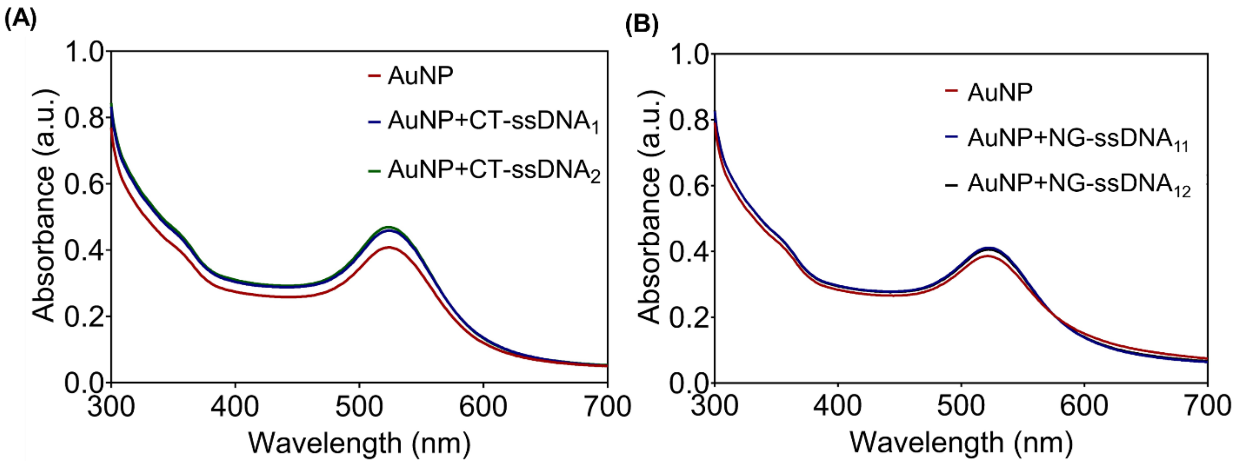

Figure S1. UV-Visible absorbance spectra of gold nanoparticles conjugated with (A) CT-ssDNA$_1$ and CT-ssDNA$_2$; (B) NG-ssDNA$_{11}$ and NG-ssDNA$_{12}$ compared to the citrate stabilized no-DNA conjugated AuNPs. The absorbance measurements were recorded from three (n=3) such independent experiments. The plots were presented using the mean value obtained after repeated measures (n=3).

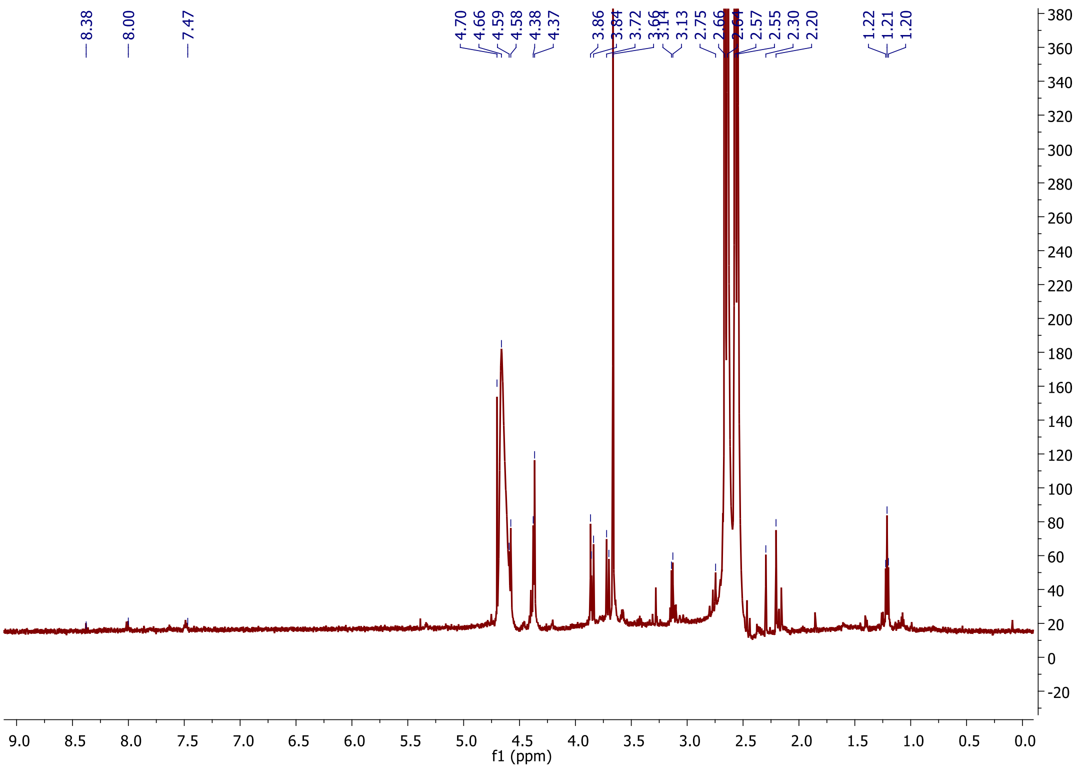

Figure S2. ^{1}H-NMR spectra of gold nanoparticles conjugated with CT-ssDNA$_1$.

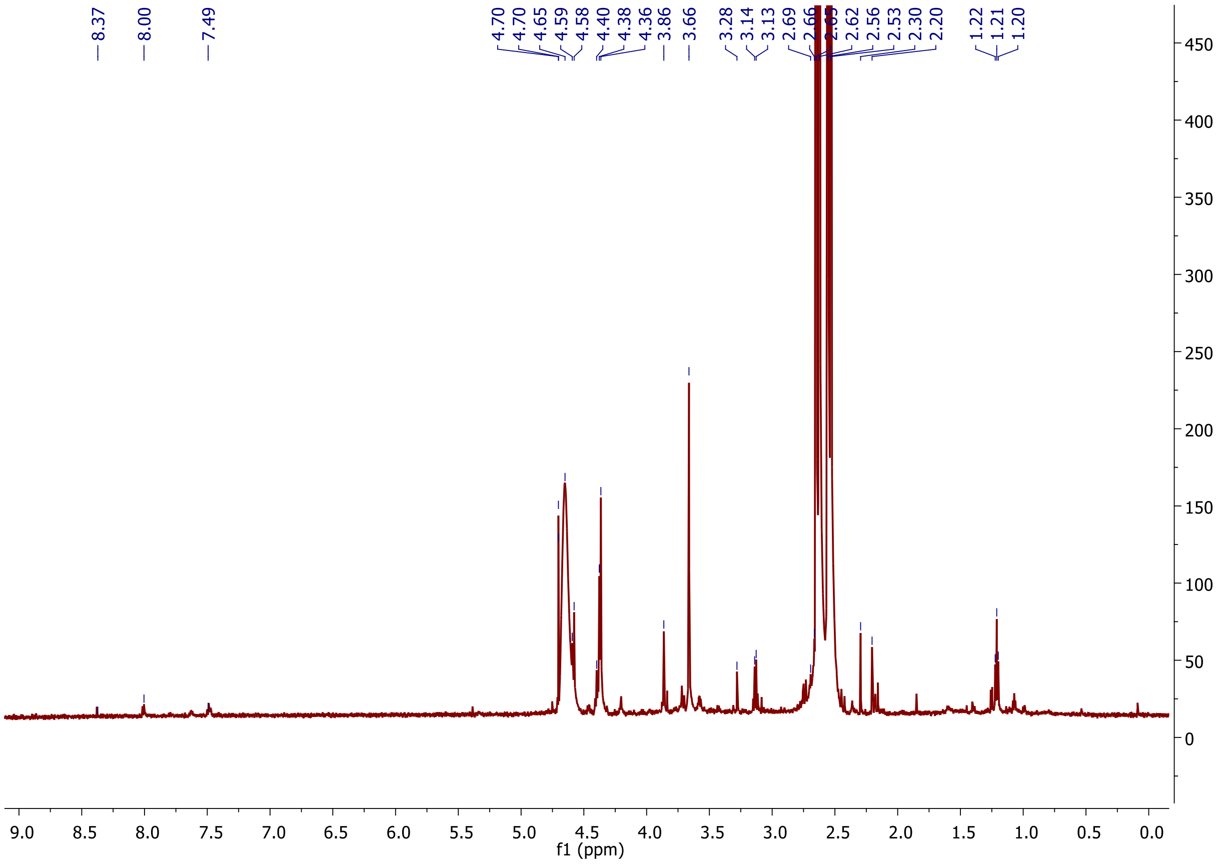

Figure S3. ^{1}H-NMR spectra of gold nanoparticles conjugated with NG-ssDNA$_{11}$.

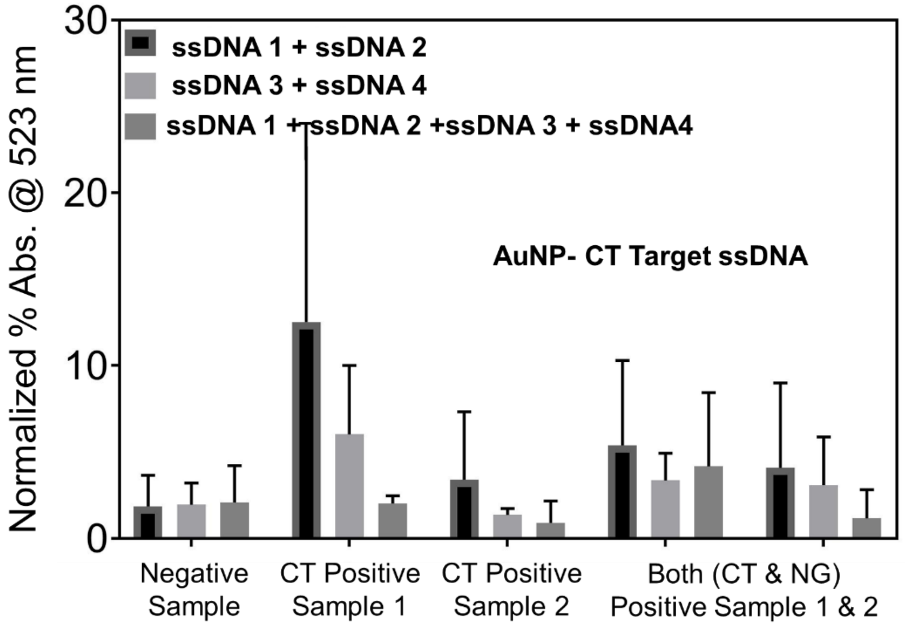

Figure S4. The normalized absorbance at 523 nm for gold nanoparticles conjugated with CT targeted ssDNAs in presence of DNA extracted from CT positive, CT and NG positive and control negative samples. N=2 for both CT positive and CT and NG positive. Working concentration of the ssDNA conjugated with AuNPs was 0.5 µM. Data are presented as mean ± SD. Error bar indicates the measurements from three (n=3) such independent experiments.

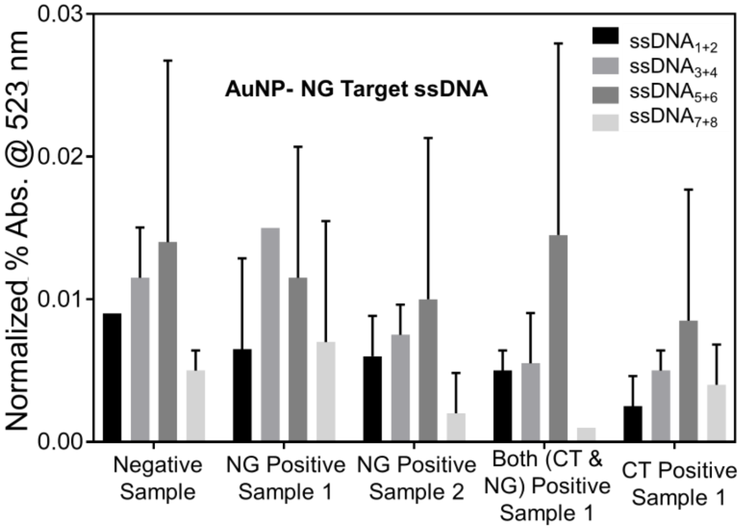

Figure S5. The normalized absorbance at 523 nm for gold nanoparticles conjugated with NG targeted ssDNAs in presence of DNA extracted from NG positive, CT and NG positive and negative control samples. N=2 for both NG positive and CT and NG positive. Working concentration of the ssDNA conjugated with AuNPs was 0.5 µM. Data are presented as mean ± SD. Error bar indicates the measurements from three (n=3) such independent experiments.

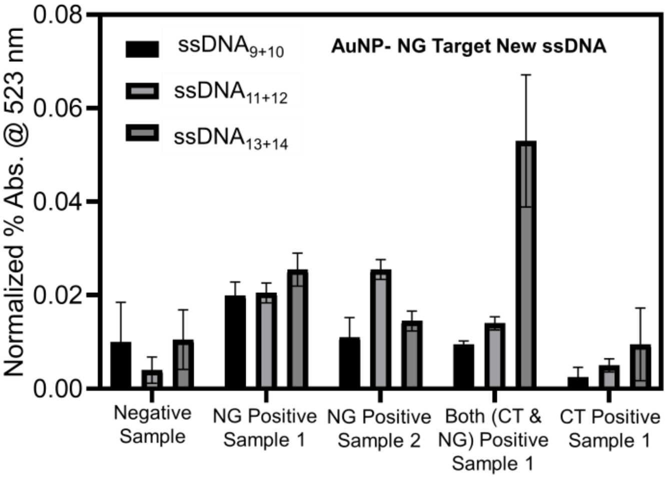

Figure S6. The normalized absorbance at 523 nm for gold nanoparticles conjugated with newly designed NG targeted ssDNAs in presence of DNA extracted from NG positive, CT and NG positive and negative control samples. N=2 for both NG positive and CT and NG positive. Working concentration of the ssDNA conjugated with AuNPs was 0.5 µM. Data are presented as mean ± SD. Error bar indicates the measurements from three (n=3) such independent experiments.

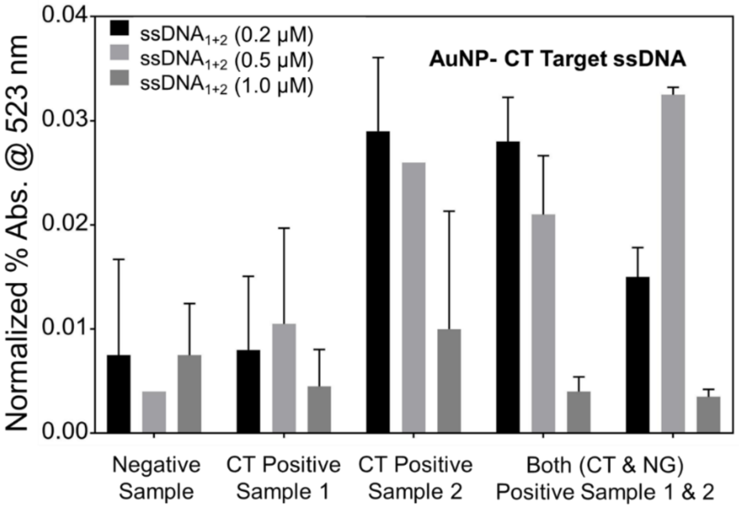

Figure S7. The normalized absorbance at 523 nm for gold nanoparticles conjugated with CT targeted ssDNAs in presence of DNA extracted from CT positive, CT and NG positive and control negative samples. N=2 for both CT positive and CT and NG positive. The working concentration of the ssDNAs was optimized at three different concentrations. $ssDNA_{1+2}$ at 0.5 µM concentration showed the best response. Data are presented as mean ± SD. Error bar indicates the measurements from three (n=3) such independent experiments.

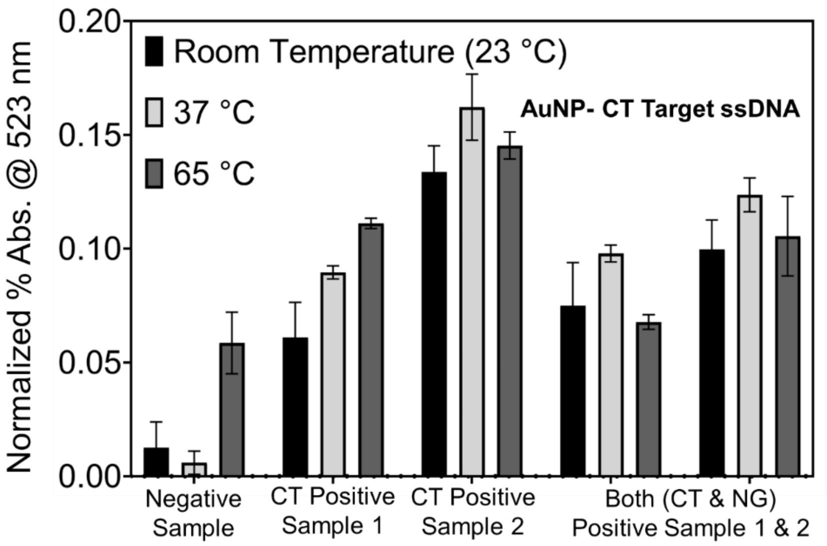

Figure S8. The normalized absorbance at 523 nm for gold nanoparticles conjugated with CT targeted ssDNAs at three different temperatures in presence of DNA extracted from CT positive, CT and NG positive and control negative samples. N=2 for both CT positive and CT and NG positive. The optimized working concentration of the ssDNAs as 0.5 µM was used for the study. It was observed that the AuNP-CT targeted ssDNAs showed optimum response at 37 °C. Data are presented as mean ± SD. Error bar indicates the measurements from three (n=3) such independent experiments.

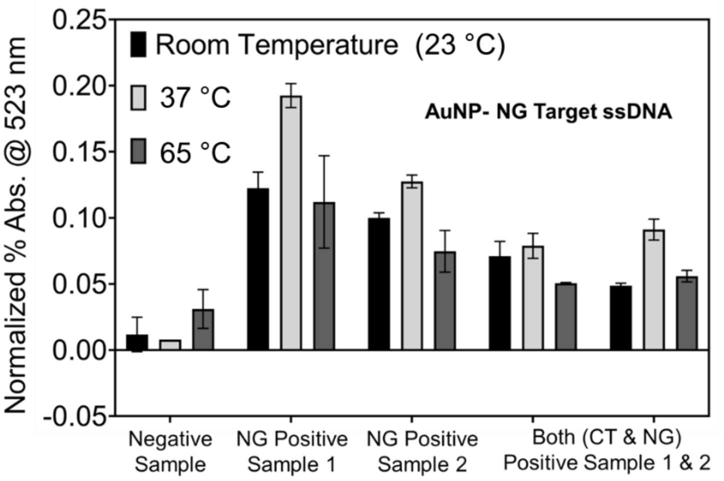

Figure S9. The normalized absorbance at 523 nm for gold nanoparticles conjugated with NG targeted ssDNAs at three different temperatures. in presence of DNA extracted from NG positive, CT and NG positive and control negative samples. N=2 for both NG positive and CT and NG positive. The optimized working concentration of the ssDNAs as 0.5 µM was used for the study. It was observed that the AuNP-NG targeted ssDNAs showed optimum response at 37 °C. Data are presented as mean ± SD. Error bar indicates the measurements from three (n=3) such independent experiments.

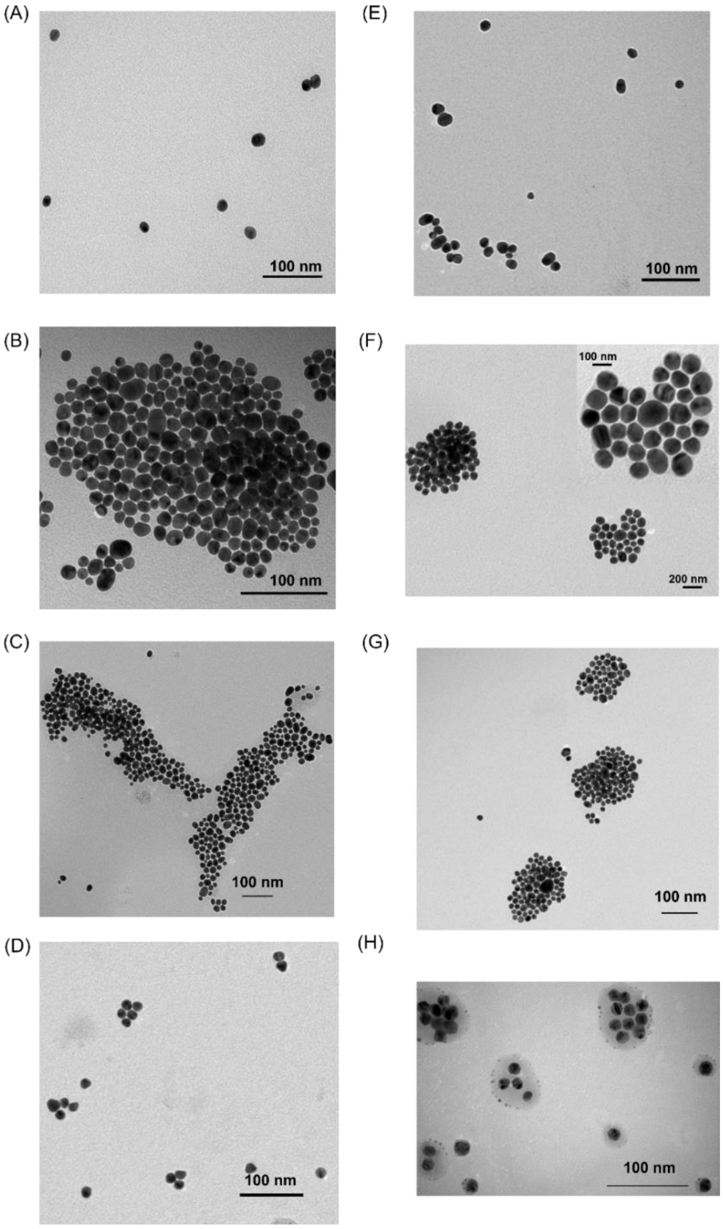

Figure S10. Agglomeration of ssDNA-capped AuNPs in the presence of bacterial DNA studied using transmission electron microscopy (TEM). (**A-D**) NG targeted ssDNAs capped with AuNPs. (**E-H**) CT targeted ssDNAs capped with AuNPs. (**A**) NG targeted ssDNAs capped with AuNPs in presence of control sample (water). No agglomeration was observed. (**B**) NG targeted ssDNAs capped with AuNPs in presence of NG positive sample. Agglomeration was observed in the presence of target NG DNA. (**C**) NG targeted ssDNAs capped with AuNPs in presence of NG+CT positive sample. Agglomeration was observed. (**D**) NG targeted ssDNAs capped with AuNPs in presence of NG negative but CT positive sample. No agglomeration was observed in the absence of target NG DNA. (**E**) CT targeted ssDNAs capped with AuNPs in presence of control sample (water). No agglomeration was observed. (**F**) CT targeted ssDNAs capped with AuNPs in presence of CT positive sample. Agglomeration was observed in the

presence of target CT DNA. (**G**) CT targeted ssDNAs capped with AuNPs in presence of NG+CT positive sample. Agglomeration was observed. (**H**) CT targeted ssDNAs capped with AuNPs in presence of CT negative but NG positive sample. No agglomeration was observed in the absence of target CT DNA.

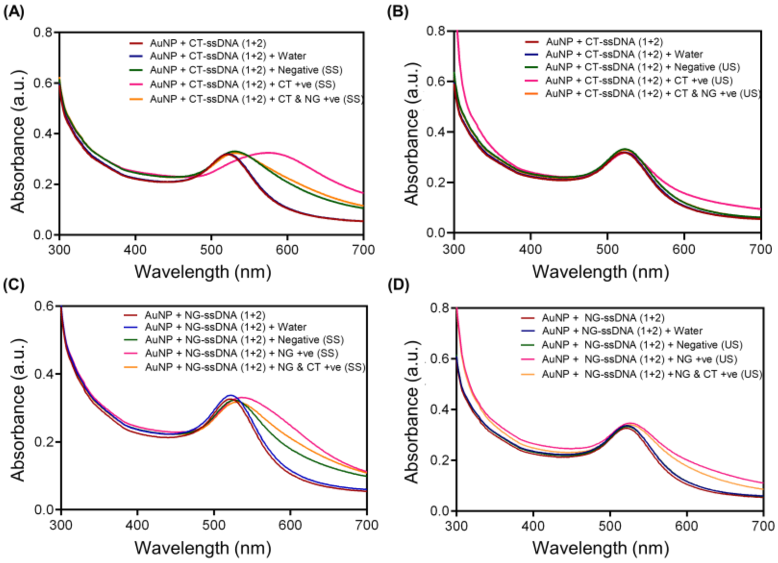

Figure S11. UV-Visible absorbance spectra of gold nanoparticles conjugated with (**A-B**) CT-ssDNAs and (**C-D**) NG-ssDNAs tested for CT DNA and NG DNA respectively. (**A**) Absorption spectra of gold nanoparticles conjugated with CT-ssDNAs when tested for CT DNA directly from cervical swab samples. Clear agglomeration at 630 nm can be observed for CT positive and both CT+NG positive samples. No agglomeration is observed when tested with negative samples. (**B**) Absorption spectra of gold nanoparticles conjugated with CT-ssDNAs when tested for CT DNA directly isolated from urine samples using CHAI buffer. Increase in agglomeration was observed for CT positive sample with an increase in absorbance at 630 nm. No agglomeration is observed when tested with negative samples. (**C**) Absorbance spectra of gold nanoparticles conjugated with NG-ssDNAs when tested for NG DNA directly isolated from cervical swab samples using CHAI buffer. Clear shift in wavelength maxima can be observed for NG positive and both CT+NG positive samples. No agglomeration is observed when tested with negative samples. (**D**) Absorbance spectra of gold nanoparticles conjugated with NG-ssDNAs when tested for NG DNA directly from urine samples. Increase in absorbance at 630 nm can be observed indicative of increase in agglomeration in presence for NG positive and both CT+NG positive samples. No agglomeration is observed when tested with negative samples. The absorbance measurements were recorded from three (n=3) such independent

experiments. The plots were presented using the mean value obtained after repeated measures (n=3).

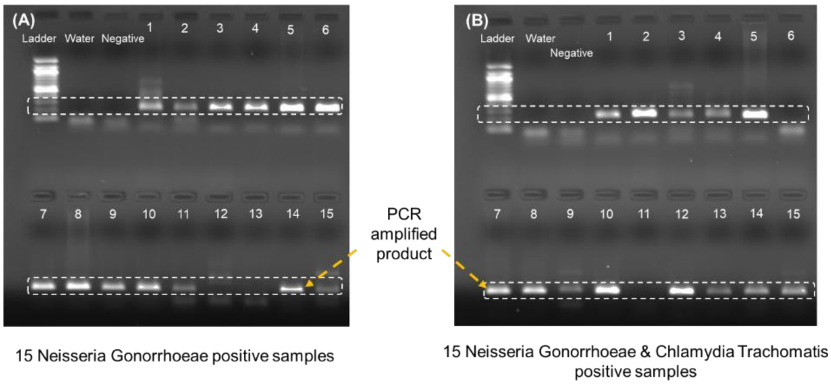

Figure S12. Agarose gel-electrophoresis results for PCR validated and amplified samples using NG DNA targeted primers. **(A)** 15 NG positive samples (1-15) along with 100bp ladder, control sample (water) and NG negative sample were run on a 2% agarose gel. **(B)** 15 both NG positive CT positive samples (1-15) along with 100bp ladder, control sample (water) and NG negative sample were run on a 2% agarose gel. Clear band was observed in most of the 15 NG positive samples but was found to be absent in the negative cases. Faint band was observed in a few of the positive cases indicative of lower DNA concentration (higher Ct value) in those samples even after PCR amplification. Sample 13 in Figure S12A along with samples 6 and 11 in Figure S12B are such samples with Ct values higher than 33.

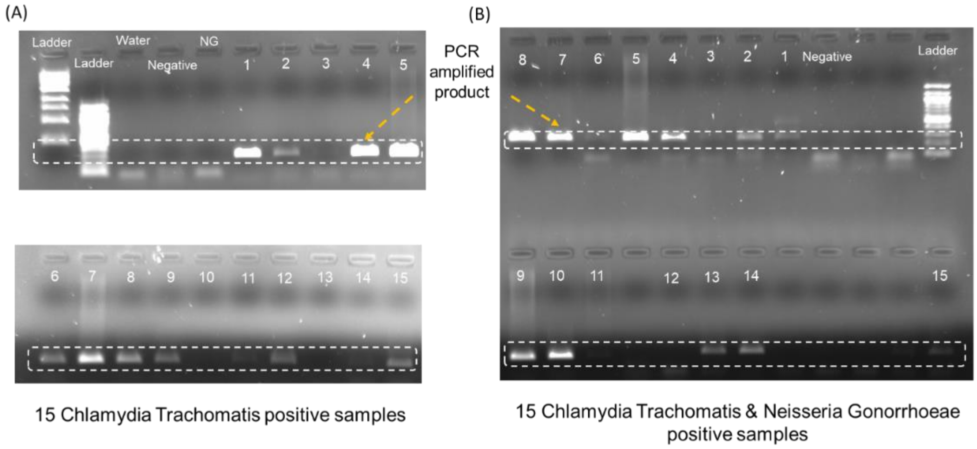

Figure S13. Agarose gel-electrophoresis results for PCR validated and amplified samples using CT DNA targeted primers. **(A)** 15 CT positive samples (1-15) along with 100 bp ladder, control sample (water), CT negative sample and NG positive sample were run on a 2% agarose gel. **(B)** 15 both NG+CT positive samples (1-15) along with 100 bp ladder, control sample (water), CT negative and NG positive sample were run on a 2% agarose gel. Clear band was observed in

most of the 15 CT positive samples on the gel but was found to be absent in other cases. Faint band was observed in a few of the positive cases indicative of lower DNA concentration (higher Ct value) in those samples even after PCR amplification. Samples 10 and 13 in Figure S13A and sample 11 in Figure S13B are such samples with Ct values higher than 33.

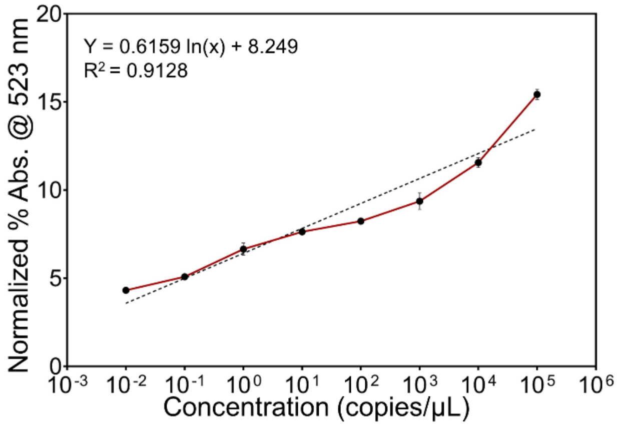

Figure S14. Analytical limit of detection for gonorrhea using NG targeted ssDNA conjugated to gold nanoparticles. The assay was tested using the genomic DNA serially diluted 10-fold times with concentrations ranging from 10^5 copies/µL to 10 copies/mL. The limit of detection was calculated using the equation $3.3 * (\frac{Sy}{S})$ where (Sy) is the standard deviation of the response of the curve and (S) is the slope of the calibration curve. The absorbance measurements were recorded from three (n=3) such independent experiments.

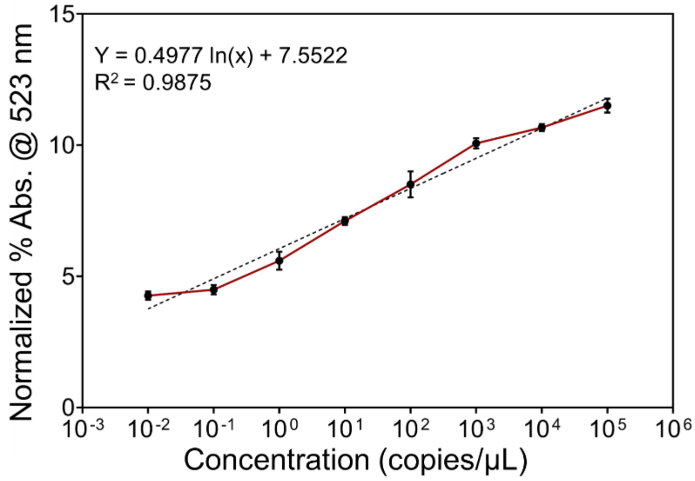

Figure S15. Analytical limit of detection for chlamydia using CT targeted ssDNA conjugated to gold nanoparticles. The assay was tested using the genomic DNA serially diluted 10-fold times with concentrations ranging from 10^5 copies/µL to 10 copies/mL. The limit of detection was calculated using the equation $3.3 * (\frac{Sy}{S})$ where (Sy) is the standard deviation of the

response of the curve and (S) is the slope of the calibration curve. The absorbance measurements were recorded from three (n=3) such independent experiments.

Targeted Towards	Target Region	Nucleotide Gap	Target Sequence (5'—3')	ssDNA (5'—3')	Numeric Convention
Cryptic Plasmid ORF6	287 – 306	26	CCAAGAGCAGCGCCUACAAC	HS-C6-GTTGTAGGCGCTGCTCTTGG	ssDNA$_1$
	332 – 351		UCAACCUGCCCAACCAGACC	GGTCTGGTTGGGCAGGTTGA-C6-SH	ssDNA$_2$
Major Outer Membrane Protein oomph	781 – 800	45	ACCAAGGACGCCAGCAUCGA	HS-C6-TCGATGCTGGCGTCCTTGGT	ssDNA$_3$
	845 – 864		ACAUGUUCACCCCCUACAUC	GATGTAGGGGGTGAACATGT-C6-SH	ssDNA$_4$

Table S1. ssDNA sequences identified towards the cryptic plasmid (ORF6) and major outer membrane protein (oomph) of *Chlamydia trachomatis*

Targeted Towards	Target Region	Nucleotide Gap	Target Sequence (5'—3')	ssDNA (5'—3')	Numeric Convention
Major Outer Membrane Protein NGK_2092	33 – 52	51	GAACGACGCCGACGACAAGA	HS-C6-TCTTGTCGTCGGCGTCGTTC	ssDNA$_1$
	103 – 122		UACGCCGGCUACACCUACAC	GTGTAGGTGTAGCCGGCGTA-C6-SH	ssDNA$_2$
Major Outer Membrane Protein NGK_2093	196 – 215	03	AACACCACCAAGACCAAGAC	HS-C6-GTCTTGGTCTTGGTGGTGTT	ssDNA$_3$
	218 – 237		ACAGCGAGAAGUUCAACGAG	CTCGTTGAACTTCTCGCTGT-C6-SH	ssDNA$_4$
Cryptic Plasmid cppA	113 – 132	29	ACCCCAGCAACAUCGCCAAG	HS-C6-CTTGGCGATGTTGCTGGGGT	ssDNA$_5$
	161 – 180		UGAAGAGGACCGAGAACGCC	GGCGTTCTCGGTCCTCTTCA-C6-SH	ssDNA$_6$
Cryptic Plasmid cppB	341 – 360	07	AGACCAAGACCAAGGGCCUG	HS-C6-CAGGCCCTTGGTCTTGGTCT	ssDNA$_7$
	367 – 386		GAGAACUGCCCCGUGGAGAA	TTCTCCACGGGGCAGTTCTC-C6-SH	ssDNA$_8$

Table S2. ssDNA sequences identified towards the cryptic plasmid (cppA & cppB) and major outer membrane protein (NGK_2092 and NGK_2093) of *Neisseria gonorrhoeae*

Targeted Towards	Target Region	Nucleotide Gap	Target Sequence (5'—3')	ssDNA (5'—3')	Numeric Convention
Major Outer Membrane Protein NGK_2093	246 – 265	27	CUUCAACUACAAAAACAGCG	HS-C6-CGCTGTTTTTGTAGTTGAAG	ssDNA$_9$
	292 – 311		UACAAACGCCACAACUACAC	GTGTAGTTGTGGCGTTTGTA-C6-SH	ssDNA$_{10}$
Major outer membrane protein NGK_2093	606 – 625	13	CGGCGAAGGCACCAAAAAAA	HS-C6-TTTTTTTGGTGCCTTCGCCG	ssDNA$_{11}$
	638 – 657		ACCAAGUCUACAGCAUCCCG	CGGGATGCTGTAGACTTGGT-C6-SH	ssDNA$_{12}$
Cryptic Plasmid cppA	118 – 137	20	AGCAACAUCGCCAAAGUCUU	HS-C6-AAGACTTTGGCGATGTTGCT	ssDNA$_{13}$
	157 – 176		GCCUUGAAACGCACCGAAAA	TTTTCGGTGCGTTTCAAGGC-C6-SH	ssDNA$_{14}$

Table S3. Another set of ssDNA sequences identified towards the cryptic plasmid (cppA) and major outer membrane protein (NGK_2093) of *Neisseria gonorrhoeae*.

Sample No.	Type	CT Value
1	CT+	26.97
2	CT+	30.08
3	CT+	29.99
4	CT+	25.30
5	CT+	0.86
6	CT+	33.22

7	CT+	31.40
8	CT+	29.09
9	CT+	30.61
10	CT+	34.25
11	CT+	24.81
12	CT+	29.14
13	CT+	35.36
14	CT+	27.84
15	CT+	25.69
16	CTNG+	29.27
17	CTNG+	29.97
18	CTNG+	30.16
19	CTNG+	30.86
20	CTNG+	23.65
21	CTNG+	25.93
22	CTNG+	22.89
23	CTNG+	19.82
24	CTNG+	27.48
25	CTNG+	23.84
26	CTNG+	33.67
27	CTNG+	30.67
28	CTNG+	28.21
29	CTNG+	22.42
30	CTNG+	29.35

Table S4. Equivalent Ct values from qPCR for *Chlamydia trachomatis* and *Chlamydia trachomatis & Neisseria Gonorrhoeae* positive samples.

Sample No.	Type	CT Value
1	NG+	26.33
2	NG+	27.78
3	NG+	19.92
4	NG+	24.16
5	NG+	20.26
6	NG+	24.20
7	NG+	23.19
8	NG+	17.98
9	NG+	23.78
10	NG+	33.19
11	NG+	34.99
12	NG+	27.89
13	NG+	35.49
14	NG+	26.94

15	NG+	31.11
16	CTNG+	27.90
17	CTNG+	21.05
18	CTNG+	32.09
19	CTNG+	27.73
20	CTNG+	18.35
21	CTNG+	33.73
22	CTNG+	24.67
23	CTNG+	23.15
24	CTNG+	25.94
25	CTNG+	19.75
26	CTNG+	35.94
27	CTNG+	33.58
28	CTNG+	21.81
29	CTNG+	28.11
30	CTNG+	25.93

Table S5. Equivalent Ct values from qPCR for *Neisseria Gonorrhoeae* and *Chlamydia trachomatis & Neisseria Gonorrhoeae* positive samples.
